# Rational engineering of *Saccharomycescerevisiae* towards improved tolerance to multiple inhibitors in lignocellulose fermentations

**DOI:** 10.1186/s13068-021-02021-w

**Published:** 2021-08-28

**Authors:** Bianca A. Brandt, Maria D. P. García-Aparicio, Johann F. Görgens, Willem H. van Zyl

**Affiliations:** 1 Department of Microbiology, Stellenbosch University, Private Bag X1, Stellenbosch, 7602 South Africa; 2 Department of Process Engineering, Stellenbosch University, Private Bag X1, Stellenbosch, 7602 South Africa

**Keywords:** Lignocellulose, *Saccharomycescerevisiae*, Microbial inhibitors, Spent sulphite liquor

## Abstract

**Background:**

The fermentation of lignocellulose hydrolysates to ethanol requires robust xylose-capable *Saccharomyces* *cerevisiae* strains able to operate in the presence of microbial inhibitory stresses. This study aimed at developing industrial *S.* *cerevisiae* strains with enhanced tolerance towards pretreatment-derived microbial inhibitors, by identifying novel gene combinations that confer resistance to multiple inhibitors (thus cumulative inhibitor resistance phenotype) with minimum impact on the xylose fermentation ability. The strategy consisted of multiple sequential delta-integrations of double-gene cassettes containing one gene conferring broad inhibitor tolerance (*ARI1*, *PAD1* or *TAL1*) coupled with an inhibitor-specific gene (*ADH6*, *FDH1* or *ICT1*). The performances of the transformants were compared with the parental strain in terms of biomass growth, ethanol yields and productivity, as well as detoxification capacities in a synthetic inhibitor cocktail, sugarcane bagasse hydrolysate as well as hardwood spent sulphite liquor.

**Results:**

The first and second round of delta-integrated transformants exhibited a trade-off between biomass and ethanol yield. Transformants showed increased inhibitor resistance phenotypes relative to parental controls specifically in fermentations with concentrated spent sulphite liquors at 40% and 80% v/v concentrations in 2% SC media. Unexpectedly, the xylose fermentation capacity of the transformants was reduced compared to the parental control, but certain combinations of genes had a minor impact (e.g. *TAL1* + *FDH1*). The *TAL1* + *ICT1* combination negatively impacted on both biomass growth and ethanol yield, which could be linked to the *ICT1* protein increasing transformant susceptibility to weak acids and temperature due to cell membrane changes.

**Conclusions:**

The integration of the selected genes was proven to increase tolerance to pretreatment inhibitors in synthetic or industrial hydrolysates, but they were limited to the fermentation of glucose. However, some gene combination sequences had a reduced impact on xylose conversion.

**Supplementary Information:**

The online version contains supplementary material available at 10.1186/s13068-021-02021-w.

## Background

The increased pressure towards decreased carbon emissions has spurred the development of lignocellulose-derived biofuels production as replacement for conventional fossil fuels [1, 2]. Fermentation serves as bioconversion to alcohols of sugar hydrolysates derived from polysaccharide-rich lignocellulose biomass. However, a major challenge linked to hydrolysis-fermentation of lignocellulose biomass is the recalcitrant nature of the material to enzymatic conversion [3, 4]. Physicochemical pre-treatment is thus required to disrupt the compact crystalline structure and allow enzymatic access to the polysaccharides within, to release fermentable sugars [3, 5, 6]. The majority of such pre-treatment methods result in significant quantities of degradation products being formed, which have inhibitory effects of subsequent biological conversions [5, 7–11].

*Saccharomycescerevisiae* cannot naturally utilize xylose, the most abundant pentose sugar within lignocellulosic hydrolysates [12–14]. Although co-fermentation of glucose and xylose remains a challenge, advances in strain development have resulted in the development of industrial *S.* *cerevisiae* strains with xylose-utilizing capacity. Through metabolic engineering, heterologous xylose catabolic pathways such as the fungal oxidoreductive pathway (XR-XDH) [13, 15, 16] or a bacterial xylose isomerase (XI) [17, 18] have been introduced into *S.* *cerevisiae* strains, as well combining both pathways into the yeast, simultaneously [14]. Recently, the industrial strain CelluX^TM^1 [19], a xylose engineered strain with a XI pathway, has been developed.

Interestingly, an unanticipated phenotype that has emerged from xylose strain development is hyper-sensitivity of the introduced heterologous metabolic pathways to stressful conditions [2]. Due to the inter-connectivity between metabolism and stress response, strain development for lignocellulose bioconversion technologies have to simultaneously address both xylose utilization and microbial stresses [10]. Xylose engineered industrial strains are thus the ideal genetic background in which to study the impact of microbial stresses, as well as introducing stress resistance genes. Compared to the XR-XDH route, there is limited knowledge on XI pathway-based xylose utilization for genetically engineered industrial *S.* *cerevisiae* [20]. Furthermore, there is a lack of studies on the interaction of genes involved in inhibitor tolerance and xylose fermentation in these yeasts.

Fermentation strains are subjected to various microbial stresses during lignocellulose bioconversion that include microbial inhibitory compounds generated during physicochemical pretreatment of lignocelluloses. The concentrations of inhibitory compounds fluctuate depending on both biomass composition and pretreatment method used [9, 11, 21]. Furans are degradation products of sugars, phenolics are derived from solubilizing lignin and weak acids such as acetic and formic acids are formed during furan degradation and/or de-acetylation of hemicellulose [5, 8, 22, 23]. Such microbial inhibitors negatively impact growth, fermentation and xylose utilization ability of yeast, which results in sub-optimal ethanol productivity and yields [9, 10, 13, 16]. Given the toxicity of these compounds, studies have been undertaken to develop yeast strains capable of not only withstanding the harsh conditions associated with lignocellulosic biomass fermentations, but to also generate ethanol yields expected in industrial processes [5, 22]. Thus, microbial inhibitor toxicity represents a bottle-neck in lignocellulosic bioethanol production and negating these detrimental inhibitory effects remains to be a fundamental challenge [22, 24, 25].

Inhibitor resistance is characterized as a complex function of multiple genes [10, 26–29], however, relatively few over-expression studies have undertaken to genetically engineer yeast towards multiple inhibitor tolerance phenotypes. Typical rational design strategies are often limited to only a few genes involved in highly specific in situ detoxification mechanisms, resulting in strains with inhibitor-specific detoxification phenotypes [30–34]. Examples include overexpression of either *TAL1*, *FDH1*, or *HAA1* for weak acids resistance [30, 35] and *ADH6*, *ADH7* or *ARI1* [36–38] for furfural detoxification mechanisms, as well as *PAD1* and/or *FDC1* that are linked to phenolic detoxification [33, 34, 39]. These studies have several shortcomings in that (i) the majority of overexpression studies are conducted in laboratory strains limiting applicability to industrial strains; (ii) strategies are limited by the available knowledge of the molecular genetic basis of resistance phenotypes gained from laboratory strains; (iii) studies make use of synthetic cocktails to simulate industrial stresses, and (iv) very few host strains are proficient xylose utilizers. Thus, more research is required to elucidate gene interaction or synergism within the dynamics of inhibitor resistance phenotypes in industrial strains towards industrial hydrolysates. A cumulative strain development strategy that combines multiple positive gene interactions from various stress response pathways could significantly enhance the yeast stress response towards multi-inhibitor resistance phenotypes. As a result, this study aims to combine inhibitor resistance pathways within a metabolic engineering approach towards the development of efficient multi-inhibitor resistant xylose-utilizing strains.

This study explored the simultaneous overexpression of multiple native gene targets that confer resistance to weak acids, furan aldehydes and phenolic compounds individually, and thereby identify gene combinations that could generate cumulative multi-inhibitor resistance phenotypes. *ARI1* (NADPH-dependent aldehyde reductase), *PAD1* (flavin prenyltransferase) and *TAL1* (transaldolase) were selected since these genes have been implicated in broader multi-inhibitor resistance phenotypes [32, 39, 40], whereas *FPS1* (aquaglyceroporin), *FDH1* (formate dehydrogenase 1), *ADH6* (NADP-dependent alcohol dehydrogenase 6) and *ICT1* (1-acylglycerol-3-phosphate *O*-acyltransferase) address specific effects attributed to weak acid [30, 41, 42], furan aldehyde [37] and organic solvent stresses [43], respectively. The partial deletion of *FPS1* and inclusion of *ICT1* allowed for the novel regulation of “membrane-modulating” genes into resistance phenotypes [44]. Dual gene combinations were constructed to have a multi-inhibitor resistance gene coupled with a target-specific gene in an *FPS1* deletion background. Not only does this approach allow for the introduction of genes in novel sequential combinations, but also the assessment of these genes in industrial strains exposed to industrial-like lignocellulose fermentations. Ultimately, this improves the current understanding of in situ detoxification of lignocellulose-derived inhibitors for the development of robust xylose-capable *S.* *cerevisiae* industrial strains.

## Results

### Chemical composition of lignocellulose hydrolysate and SSL

Table 1 lists some of the components determined in the liquid fraction of steam-exploded sugarcane bagasse (SCB), as well as SSL from MgO acid sulphite pulping process of mixed hardwood feedstocks. The pretreatment as well as sulphite pulping process resulted in the solubilization of the hemicellulosic fraction, but lignin was solubilized more intensely in the case of the sulphite process. Both liquors were composed of fermentable sugars and inhibitors such as weak acids (15.7 and 6.5 g L^−1^ for SSL and SCB hydrolysate, respectively), furans (2.3 and 2 g L^−1^ for SSL and SCB hydrolysate, respectively) and phenolics (2 g L^−1^ and 0.8 g L^−1^ for SSL and SCB hydrolysate, respectively). In terms of carbohydrates, the main sugar present was xylose with values of about 93 g L^−1^ and 8.7 g L^−1^ for the SSL and SCB hydrolysate, respectively. Literature reports xylose as the main sugar present in SSL liquors generated from hardwood feedstocks (HSSL) [11]. Also, SSL contained considerably more xylose than SCB hydrolysate, as well as significant concentrations of inhibitors, especially acetic acid (15.1 g L^−1^, about 3 times more than SCB hydrolysate) and phenolic compounds (2 g L^−1^, two times more than in steam explosion hydrolysate). Moreover, the SSL also has other compounds that can act as inhibitors such as SO_2_ (used extensively in the wine industry to limit microbial contamination at lower pH values) and MgO (inhibits the growth of *S.* *cerevisiae)*, making hardwood SSL a particularly challenging lignocellulosic feedstock [11].

**Table 1:** Chemical composition of concentrated hardwood-SSL and sugarcane hydrolysate

Component	Hardwood SSL	Hydrolysate–pretreatment liquor	Units
Glucose	14.7 ± 0.02	1.04 ± 0.02	g L^−1^
Xylose	92.7 ± 1.03	8.70 ± 0.06	g L^−1^
Furfural	2.08 ± 0.00	1.66 ± 0.01	g L^−1^
HMF^a^	0.21 ± 0.01	0.35 ± 0.00	g L^−1^
Acetic acid	15.1 ± 0.48	5.76 ± 0.02	g L^−1^
Formic acid	0.56 ± 0.01	0.74 ± 0.02	g L^−1^
Cinnamic acid	72.0 ± 18.0	–	mg L^−1^
Ferulic acid	275 ± 21.4	33.8 ± 4.80	mg L^−1^
3,4-DHBA^b^	46.1 ± 9.25	516 ± 4.22	mg L^−1^
3–5 DHBA^b^	1.05 ± 0.36	0.002 ± 0.39	g L^−1^
Vanillic acid	116 ± 32.1	16.1 ± 0.56	mg L^−1^
Syringic acid	308 ± 34.4	29.3 ± 0.23	mg L^−1^
Vanillin	76.1 ± 9.39	135 ± 3.05	mg L^−1^
Syringaldehyde	138 ± 14.1	23.1 ± 1.15	mg L^−1^
Coniferaldehyde	15.9 ± 6.20	16.6 ± 0.77	mg L^−1^
MgO	17.2	–	g L^−1^
SO_2_	0.6	–	g L^−1^

^a^Hydroxymethylfurfural

^b^Dihydroxybenzoic acid

### Strain development in lignocellulose hydrolysate and inhibitor tolerance assays

The multi-inhibitor-resistant strain construction strategy centred on three rounds of sequential delta integration of double-gene expression cassettes to construct strains overexpressing selected inhibitor resistance genes in different combinations. Overexpression of respective double-gene cassettes was facilitated by homologous recombination of delta-integration cassettes into native delta sequences distributed in the parental CelluX^TM^1 yeast genome. Transformants were screened for growth and ethanol yield in lignocellulosic hydrolysate after each round of integration to select the best strains for the next round of transformation, with final transformants assayed for inhibitor tolerance phenotypes.

Before the integration of inhibitor tolerance genes commenced, a partial *FPS1* deletion variant of *S.* *cerevisiae* CelluX^TM^1 was generated. Eight partial *FPS1* deletion transformants were selected by screening for higher ethanol yield (g ethanol g^−1^ total sugar) on 2% YPD supplemented with 65% v/v sugarcane hydrolysate (Table 1). The best performing transformant, CelluX1∆*FPS1-*C5, yielded 0.41 g g^−1^ at 169 h that showed a 5% ethanol yield increment to the parental CelluX^TM^1 strain at 0.39 g g^−1^ (see Additional file 1: Tables S2, S3). Interestingly, strain CelluX1∆*FPS1-*C5 also exhibited an increase of 19.8% in formic acid detoxification and higher xylose consumption at 53.6% compared to 49.8% of the parental strain. This partial *FPS1* deletion *CelluX1*∆*FPS1-*C5 strain was used as the host for the first round of delta integrations.

The results from the best transformant per gene combination from the first round of delta integration are listed in Table 2. The first round of transformants often exhibited a trade-off between growth and ethanol yield (see Additional file 2: Figure S1). The pBKD-AA integration cassette with the *ARI1* and *ADH6* genes that confer furan resistance, in combination with partial *FPS1* deletion allowed for furan and weak acid resistance*.* Final ethanol yields of pBKD-AA transformants showed overall improvement that ranged from 0.34 to 0.38 g g^−1^ as compared to the parental CelluX^TM^1 strain at 0.33 g g^−1^, with a maximum increase in the ethanol yield of 15.8% (Table 2). Partial deletion of the *FPS1* gene in combination with pBKD-AA integrations proved beneficial to inhibitor resistance phenotype in terms of cell growth, as 80% of transformants displayed similar or increased growth (measured in absorbance) compared to the parental strain. The pBKD-AF integration cassette overexpressing the *ARI1* and *FDH1* genes also conferred furan and weak acid resistance. This configuration, however, significantly decreased the ethanol yield by 4.29–17.2%, compared to the parental strain, although an improvement in growth was seen with AF10, showing an increment of 7.92% over the parental strain. The pBKD-AI integration cassette overexpressing the *ARI1* and *ICT1* genes conferred furan, organic solvent and weak acid resistance however, 62.5% of transformants with this configuration exhibited decreased growth of 12–15%. Likewise, ethanol yields decreased by 1–7% showing improvement for only one strain (AI1) with an increment of 1.25% over the parental reference, respectively (see Additional file 2: Figure S1).

**Table 2:** Performance of the best transformant per gene combination after 1st stage of strain development

Gene combination	Strain	Resistance phenotype	EtOH yield % increment	Growth (OD_600_) % increment
*ARI* + *ADH6*	AA6	Furans + weak acids	15.80	19.50
*ARI1* + *FDH1*	AF10	Furans + weak acids	− 5.55	7.92
*ARI1* + *ICT1*	AI1	Furans + weak acids + organic solvents	1.25	− 12.00
*PAD1* + *ADH6*	PA7	Phenolics + furans + weak acids	− 1.97	3.40
*PAD1* + *FDH1*	PF5	Phenolics + weak acids	− 15.40	31.80
*PAD1* + *ICT1*	PI3	Phenolics + weak acids + organic solvents	− 3.58	20.90
*TAL1* + *ADH6*	TA6	Furans + weak acids	3.60	18.60
*TAL1* + *FDH1*	TF2	Weak acids	16.90	6.20
*TAL1* + *ICT1*	TI10	Weak acids + organic solvents	− 8.34	− 22.30

The pBZD-PA integration cassette overexpressing the *PAD1* and *ADH6* genes and this configuration conferred resistance to furans, phenolics and weak acids. After the first round of delta integrations, the ethanol yields of the transformants were lower by 2–24.6% or equal to that of the parental strain with six transformants showing improved growth by 1–18.8% growth increment (see Additional file 2: Figure S1). The pBZD-PF cassette integration overexpresses the *PAD1* and *FDH1* genes that confer resistance to phenolics and weak acids. These transformants also exhibited no increases in ethanol yields, but showed an increase in growth by 5–31.7% for most of the transformants. A similar trend was observed in the transformants with the pBZD-PI integration cassette overexpressing the *PAD1* and *ICT1* genes*,* which confers resistance to weak acids, phenolics and organic solvents. Ethanol yields were either lower or similar to the parental strain, whereas growth was either similar or higher than that of the reference strain with PI3 transformant showing a maximum growth increment of 20.9%.

The pBHD-TA integration cassette overexpresses the *TAL1* and *ADH6* genes that confer furan and weak acid resistance. After the first round of delta integrations, the pBHD-TA transformants showed no real differences in ethanol yields relative to the parental strain with only TA6 showing improvement at 3.6% ethanol yield increment and final growth increment of 18.6%. Transformants with the pBHD-TF integration cassette overexpressing the *TAL1* and *FDH1* genes conferring weak acid resistance exhibited similar or higher ethanol yields relative to parental strain with TF2 showing the highest increment in yield at 16.9%. The pBHD-TI integration cassette overexpressing the *TAL1* and *ICT1* genes that confers resistance to weak acids and organic solvents proved to be detrimental to both the ethanol yield and growth as transformants exhibited significant decreases in both ethanol yield (8.4–35.2%) and growth (20.4–38.2%).

After the first stage of delta integration, the pBHD-TF and pBKD-AA integration cassettes generated transformants with more than 10% increment on ethanol yield relative to the parental strain. Therefore, these combinations were selected to continue into the second round that involved the integration of the pBHD-TF and pBKD-AA integration cassettes into *S.* *cerevisiae* AA6 (resulting in ATF transformants) and TF2 (TFA transformants), respectively. The second round of transformants also resulted in a trade-off between growth and ethanol yield (see Additional file 2: Figure S2). Compared to the parental strain, 70.8% of the ATF transformants favoured growth with ATF13 showing an improved growth increment at 17.3%. Conversely, 58.3% of the TFA transformants exhibited increased ethanol yields. Interestingly, when the concentration of hydrolysate in YPDX reduced from 65% v/v to 50% v/v, no significant differences were observed in resistance phenotypes between parental strain and transformants, indicating possible phenotypic plasticity in transformants.

**Fig. 1:**
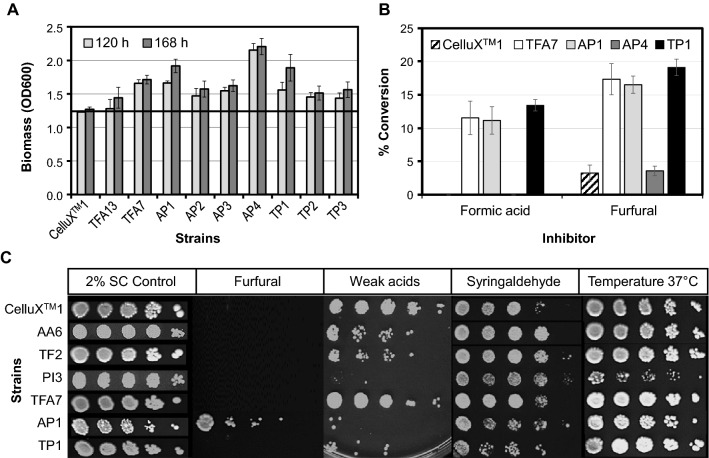
The performance of final transformants in **A** 2% SC-X supplemented with 65% v/v sugarcane hydrolysate spiked with 20 g L^−1^ furfural and formic acid, with a straight line indicating benchmark performance of CelluX^TM^1 parent and **B** the % conversion of inhibitors. **C** A spot chart illustrates transformant performance to various inhibitors/stressors at OD_600_ (from left to right) 5,1,0.5, 0.1 and 0.01 in 2% SC only or supplemented with 1 g L^−1^ furfural, 6 g L^−1^ acetic acid and 0.8 g L^−1^ formic acid, or 0.6 g L^−1^ syringaldehyde, and growth at 37 °C

In the third and final round of delta integration, plasmid pBZD-PI was integrated into the ATF13 (AP transformants) and TFA7 (TP transformants) strains, thus strains overexpress six genes in the *FPS1* partial deletion background. Subsequent transformants exhibit resistance to weak acids, furans and phenolic compounds. The strains were evaluated on growth (absorbance), fermentation ability (ethanol yield) and inhibitor detoxification (% conversion). *S.* *cerevisiae* CelluX^TM^1 was used as an industrial and parental reference strain, whereas ATF13 and TFA7 were used as additional parental reference strains. All transformants showed a significant improvement in growth compared to the parental CelluX^TM^1 strain, during fermentations with 2% SC-X media supplemented with 65% v/v sugarcane hydrolysate at pH 5 and spiked with 20 g L^−1^ of furfural and 20 g L^−1^ of formic acid (Fig. 1A). The growth profiles of the AP and TP transformants surpassed the performance of the industrial CelluX^TM^1 strain. Interestingly, TFA7 with only two gene cassettes did as well as the final transformants. AP1 and AP4 showed the highest growth (OD_600_), whereas TP1 was the best performer from the TP transformants (Fig. 1A). Only AP1 and TP1 showed a significant difference in growth between 120 h versus 168 h. As expected, ethanol concentrations were very low, ranging from 1.6 to 2.4 g L^−1^ given the extreme toxicity of fermentation media (data not shown).

The transformants showed a noteworthy difference in inhibitor detoxification phenotypes for formic acid (present as formate in the medium at pH 5) and furfural (Fig. 1B). Although AP4 showed the highest OD_600_ at 168 h, the growth could not be linked back to an improved inhibitor resistance phenotype. In contrast, the TP1 strain showed the highest detoxification phenotype with an average of 13.4% and 19% conversion of formic acid (formate) and furfural, respectively, at 168 h (Fig. 1B). CelluX^TM^1 as parental and industrial reference strain, however, showed poor growth and poor inhibitor detoxification with no formic acid converted and only 3% of the furfural detoxified. TFA7 and AP1 transformants also surpassed the parental and industrial reference strain with improved detoxification phenotypes.

The copy number of the gene inserts were determined via qPCR analysis. The TFA7, TP1 and AP1 transformants each have 1 insert of the TF (*TAL1* + *FDH1*) and AA (*ARI1* + *ADH6*) double-gene constructs, i.e. 1 additional copy of the native *TAL1*, *FDH1*, *ARI1* and *ADH6* genes. Both TP1 and AP1 transformants also have 6 additional copies of native *PAD1* and *ICT1* genes (see Additional file 1: Table S4). The copies numbers of the inserts between the transformants were similar. However, the observed phenotypes of the TFA7, TP1 and AP1 transformants were different as the AP1 strain performed poorly compared to the more robust TFA7 and TP1 strains.

Two different assay methods were applied to evaluate inhibitor tolerance phenotypes, i.e. inhibitor tolerance plate assays (pH 4.0–4.5, no pH control) and enzymatic assays. Plate assays showed variations between inhibitor phenotypes within the three stages of strain development (Fig. 1C). In particular, the PI3 strain shows increased susceptibility to weak acid stress (6 g L^−1^ acetic acid and 0.8 g L^−1^ formic acid) when no pH control was implemented (pH < 5) and this phenotype was confirmed in AP1 and TP1 transformants with pBZD-PI inserts in the third round of delta integrations (Fig. 1C), although this integration improved resistance to the phenolic syringaldehyde. AP1 was the only transformant showing resistance to 1 g L^−1^ furfural when critical mass was present [45]. The in vitro activities of detoxification enzymes were assayed to determine inhibitor detoxification potential of transformants. Detoxification was measured as the decrease in substrate, i.e. furfural, cinnamic acid or formic acid due to enzymatic degradation (see Additional file 1: Table S5). No significant differences were observed between transformants and parental reference in furfural assays. In the cinnamic acid assays, PI3 and TP1 transformants exhibited enhanced in vivo cinnamic acid detoxification activity. Similarly, formic assays showed AP1 and TP1 transformants to have enhanced formic acid detoxification phenotypes, relative to control.

### Detoxification phenotypes in simulated/synthetic inhibitor cocktail fermentations

The TFA7, AP1 and TP1 transformants were subjected to fermentations in 2% SC-X media supplemented with either, 5 g L^−1^ furfural plus 0.5 g L^−1^ HMF, 6 g L^−1^ acetic—plus 0.81 g L^−1^ formic acid, or 1 g L^−1^ cinnamic acid to ascertain detoxification phenotypes of the gene combinations to specific microbial inhibitors groups. The *S*. *cerevisiae* CelluX^TM^1 strain was used as an industrial and parental reference. The transformants from the first round of integration (AA6, TF2 and PI3) were used as secondary controls to determine if second and third-round delta-integrated transformants also exhibit phenotype from first integrations, i.e. cumulative phenotypes.

**Fig. 2:**
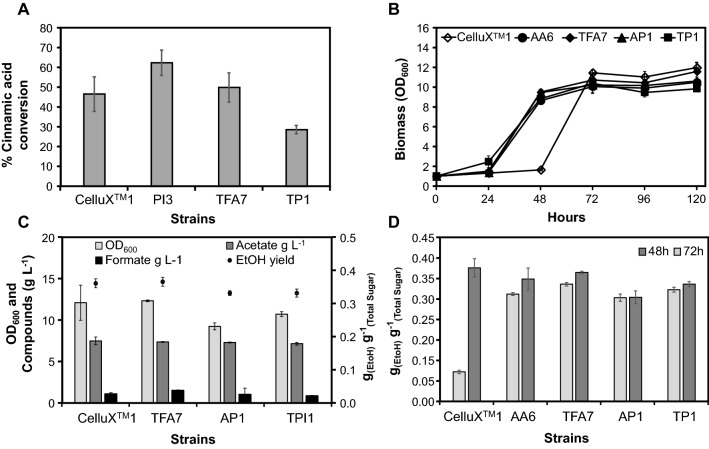
Performances of final transformants in 2% SC-X fermentations supplemented with either, **A** 1 g L^−1^ cinnamic acid, **B** 5 g L^−1^ furfural or **C** 6 g L^−1^ acetic and 1 g L^−1^ formic acid. **D** The ethanol yields of various strains in 2% SC-X fermentations supplemented with 5 g L^−1^ furfural at 48 versus 72 h

In fermentations with 1 g L^−1^ cinnamic acid, there were no differences observed between parental CelluX^TM^1 strain and TFA7 or TP1 transformants. However, the PI3 transformant exhibited an enhanced cinnamic acid detoxification phenotypes (Fig. 2A). The AA6, TFA7, AP1 and TP1 transformants showed marked improvement in furfural detoxification phenotype when compared to parental and industrial reference strains. All transformants exhibited a decrease in the lag phase of 24 h compared to the 48 h for the parental strain, with furfural detoxified within the said time period (Fig. 2B). At 48 h, transformants exhibited ethanol yields ranging from 0.25 to 0.29 g g^−1^, whereas the parental control fermentation yield was below 0.1 g g^−1^ (Fig. 2D). Glucose was depleted within 48 h versus 72 h for the parental CelluX^TM^1 reference strain with no significant differences in ethanol yield for TFA1 and AA6 strains versus the reference strain at 72 h. No significant differences in CelluX^TM^1 and TFA7 fermentation performances were observed with weak acid exposure, however, this fermentation confirmed AP1 and TP1 strains are more susceptible to weak acids due to the pBZD-PI insert (Fig. 2C).

**Fig. 3:**
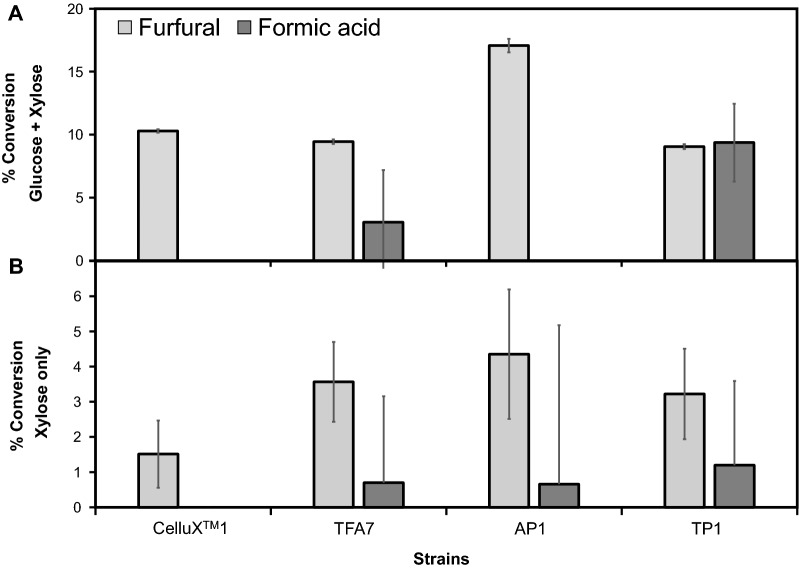
The % conversion of 5 g L^−1^ furfural and 0.8 g L^−1^ formic acid by transformants in 2% SC fermentations supplemented with synthetic IC with **A** glucose and xylose as carbon source or **B** xylose as only carbon source at 120 h

Fermentations with inhibitor cocktail were conducted with 2% SC supplemented with an inhibitor cocktail (IC) based on the composition of SSL (Table 1). Blank media supplemented with the cocktail was used as control to account for the evaporation of volatiles. Two different carbon sources were used to determine the possible effect the carbon source may have on resistance phenotypes, given the sensitivity of the introduced heterologous pathways to fermentation stresses. As anticipated, strains showed a significant difference in observed inhibitor resistance phenotypes in fermentations with glucose and xylose versus xylose only as carbon source (Fig. 3). Transformants in xylose-only fermentations showed poor detoxification phenotypes with < 5% of inhibitor compounds detoxified. In fermentations with both glucose and xylose, differences in detoxification phenotypes between transformants and the CelluX^TM^1 reference strain were observed. The AP1 transformant outperformed strain CelluX^TM^1 for furfural detoxification, whereas the TP1 transformant outperformed both AP1 and CelluX^TM^1 for formic acid detoxification. Overall, transformants exhibit enhanced detoxification phenotypes compared to CelluX^TM^1 parent.

### Hardwood SSL fermentations and final growth rate of transformants

Various concentrations of untreated SSL were used to characterize strain performances in lignocellulose fermentations in terms of consumption of glucose and xylose, ethanol concentration, ethanol yield and ethanol productivity (Table 3). In 2% SC media supplemented with 40% v/v concentrated SSL at pH 5.0, glucose was depleted within 72 h, however, xylose consumption was less than 10% for all strains with the AP1 transformant showing no xylose consumption. Ethanol yields at 72 h showed that the TP1 transformant with a yield of 0.255 g g^−1^ performed better than the CelluX^TM^1 strain with yield a of 0.228 g g^−1^ (Table 3), an 11.8% increment in yield above parental control. In 2% SC supplemented with 80% v/v concentrated SSL at pH 4.5, both parental and transformant strains showed no growth, however, strains appeared to be metabolically active as seen by the consumption of sugars (Table 3). Glucose consumption for all strains exceeded 10%, with CelluX^TM^1, TFA7 and TP1 consuming 15.1%, 15.8% and 16.3%, respectively. However, ethanol concentrations remained below 1 g L^−1^, with only CelluX^TM^1, TFA7 and TP1 strains producing ethanol, at 0.51, 0.66 and 0.48 g L^−1^, respectively.

**Table 3:** Fermentation kinetic parameters of recombinant *S.* *cerevisiae* strains and control strains

Strain	Glucose cons. %		Xylose cons. %		Ethanol						Metabolic yield %		Media pH		µMax (h^−1^)
					g L^−1^		*Y*_P/S_ g g^−1^		g L^−1^ h^−1^						
YPDX _*t*=24 h_															
CelluX^TM^1	100		100		17.0 ± 0.13		0.424 ± 0.00		0.707 ± 0.01		82.8		5		0.572
TFA7	100		100		17.1 ± 0.13		0.428 ± 0.00		0.713 ± 0.01		83.5		5		0.557
AP1	100		45.7		12.2 ± 0.09		0.305 ± 0.00		0.508 ± 0.00		82.1		5		0.513
TP1	100		100		17.3 ± 0.08		0.432 ± 0.00		0.720 ± 0.00		84.4		5		0.545
YPX _*t*=24 h_															
CelluX^TM^1	–		100		8.18 ± 0.05		0.396 ± 0.02		0.341 ± 0.00		77.7		5		0.412
TFA7	–		90.1		7.52 ± 0.25		0.364 ± 0.01		0.313 ± 0.01		78.3		5		0.364
AP1	–		42.3		3.81 ± 0.02		0.184 ± 0.00		0.159 ± 0.00		73.7		5		0.442
TP1	–		88.5		7.47 ± 0.35		0.362 ± 0.02		0.311 ± 0.01		78.9		5		0.372
40% v/v SSL _*t*=72 h_															
CelluX^TM^1	100		5.8		11.6 ± 0.93		0.228 ± 0.00		0.161 ± 0.01		–		5		–
TFA7	100		3.0		11.7 ± 0.71		0.225 ± 0.01		0.162 ± 0.01		–		5		–
AP1	100		0		11.8 ± 1.06		0.239 ± 0.00		0.160 ± 0.02		–		5		–
TP1	100		1.28		12.2 ± 1.17		0.255 ± 0.03		0.169 ± 0.02		–		5		–
80% v/v SSL _*t*=241 h_															
CelluX^TM^1	15.1		1.74		0.51 ± 0.03		n.d.^a^		–		–		4.5		–
TFA7	15.8		1.88		0.66 ± 0.03		n.d.^a^		–		–		4.5		–
AP1	10.6		0.76		n.d.^a^		–		–		–		4.5		–
TP1	16.3		1.55		0.48 ± 0.05		n.d.^a^		–		–		4.5		–

^a^Not detected/determined

The growth kinetics of the final transformants were characterized in 2% YPDX and YPX at pH 5.0 in terms of consumption of glucose and xylose, ethanol concentration, ethanol yield, ethanol productivity, metabolic ethanol yield and the maximum specific growth rate (µmax) (Table 3). In 2% YPDX, the TFA7 and TP1 transformants exhibited both increased ethanol yield and productivity at 0.428 g g^−1^ and 0.713 g L^−1^ h^−1^ and 0.432 g g^−1^ and 0.720 g L^−1^ h^−1^, respectively. The co-fermentation of glucose and xylose was only reduced for strain API1 during YPDX fermentations, unlike the low xylose utilization for all strains seen in SSL (0 – 6% xylose consumption), highlighting the pronounced effect of inhibitors on % xylose consumption. Furthermore, xylose consumption in 2% YPX decreased from 100% of the reference CelluX^TM^1 strain to 42.3 – 90.1% for transformants, indicating that the strain modification impacted negatively on xylose consumption. This was confirmed in hardwood-SSL fermentations with xylose as the main carbon source, where transformants exhibited lower % xylose consumption versus reference CelluX^TM^1 strain.

## Discussion

Efficient carbohydrate bioconversion of lignocellulose hydrolysates remains a challenge given the numerous microbial stresses and inhibitory compounds produced during pre-treatment, despite the progress on the development of industrial yeast strains with the ability of co-fermenting glucose and xylose. Overexpression of genes involved in detoxification of inhibitors could be beneficial for improving yeast tolerance. This study used a rational engineering strategy based on sequential delta homologous integrations of dual expression cassettes, allowing the introduction of several copies into the genome of the yeast. Novel target gene combinations were selected to not only modulate the cell plasma membrane against inhibitor influx, but also to direct intracellular detoxification of inhibitors and strengthen carbon/xylose metabolism. One copy of the *FPS1* gene in a xylose-capable (XI) industrial strain *S.* *cerevisiae* CelluX^TM^1 was deleted, whereas the *TAL1*, *PAD1*, *FDH1*, *ICT1*, *ARI1* and *ADH6* genes were overexpressed to develop a range of multi-inhibitor-resistant strains. The impact of gene combinations on the development of cumulative inhibitor resistance phenotypes was evaluated.

The first step of our study was the disruption of the *FPS1* aquaglyceroporin gene, which encodes a channel protein responsible for glycerol efflux and intake of acetic acid. The deletion of *FPS1* in industrial *S.* *cerevisiae* strains has proven to substantially increase both growth and ethanol yield (10–45% improvement) on glucose media under acetic acid stresses [41] and improve xylose fermentation as seen by 3–10% improvement on ethanol yield [42]. The partial deletion of the *FPS1* gene in our study, however, resulted in a moderate increase on the ethanol yield (~ 5% increment), that could be ascribed to the partial deletion and/or differences in the genetic background of the strain and media (carbon source: glucose and xylose; concentration of acetic acid 2.9 g/L, pH 5).

A CelluX1∆*FPS1-*C5 transformant was used as recipient strain for the sequential delta integration of nine different gene combinations. These combinations were assessed for ethanol yield and biomass formation (growth measured at OD_600_) during fermentations supplemented with 65% v/v sugarcane bagasse hydrolysate (Table 1). Compared to parental strains, there was generally a trade-off between the growth and the ethanol yield of the transformants from the first round of delta integrations (see Additional file 2: Figure S1). Nevertheless, some combinations of genes resulted in significant improvement on ethanol yield (*ARI1* + *ADH6* and *TAL1* + *FDH1*) while other combinations were detrimental for both parameters (*TAL1* + *ICT1*) (Table 1). The benefits of *ARI1* and *ADH6* overexpression on biomass and cell viability have been documented [37, 46]. Similarly, our results are in line with those of Sanda et al. [47] where the overexpression of both *TAL1* and *FDH1* resulted in improved ethanol production in xylose-fermenting laboratory strains.

None of the transformants screened could ferment the sugars when the SCB hydrolysate concentration in the media was increased from 65 to 75% v/v (data not shown), that suggests selecting 2–3 genes to improve inhibitor resistance fell short of multi-inhibitor resistance phenotypes. Although, it is interesting to note that the inhibitor-specific combinations AA (*ARI1* + *ADH6*) for furans and TF (*TAL1* + *FDH1*) for weak acids were the only combinations to improve both ethanol yields and biomass in 65% v/v hydrolysate fermentations. Inhibitor-specific combinations with different specificities may have a cumulative effect in constructing multi-resistance phenotypes. The AA and TF combinations were used in subsequent strain development, which resulted in strains with these specific gene combinations, but in alternate integration sequences. The PI (*PAD1* + *ICT1*) combination was included only for the final round of integrations as it did not improve ethanol yields but did improve growth (Table 2).

The second round of delta integrations also resulted in a trade-off between ethanol yield and biomass growth, with the ATF transformants more prone towards biomass (2–17% increment in 70.8% of the transformants) while the TFA transformants were more inclined to ethanol yield improvement (5–24% increment in 58.3% of the transformants) (see Additional file 2: Figure S2). It was also found that transformants presented phenotypic plasticity, with no differences in growth or ethanol yield compared to parental strain when the toxicity of the media was reduced. The selected transformant for the next round of integration, TFA7, was also able to grow in the presence of syringaldehyde at low cells concentration (Fig. 2C).

The third round of delta integration resulted in transformants with different combinations of the selected six genes in a partial *FPS1* deletion background. Of particular interest, was whether subsequent additions of inhibitor-specific gene combinations had a cumulative effect or build-up towards multi-inhibitor resistance phenotypes. The performance of the final transformants was evaluated during fermentations supplemented with inhibitors (single inhibitor or in a cocktail) and different source of sugars. The transformants were subjected to fermentations with synthetic inhibitor cocktails, as this allowed for a more controlled assessment to determine if phenotypes for specific inhibitor resistance could be linked to specific gene combinations. Compared to the parental strain, these transformants showed improved growth during fermentations supplemented with 65% v/v hydrolysate (Fig. 1A) and proved to be able to detoxify furfural and formic acid despite their high concentration in the media (20 g/L) (Fig. 1B). Final transformants presented strong furan resistance phenotypes with a 24-h reduction of the lag phase in synthetic media containing only furfural (Fig. 2B), and improved conversion of furfural when the fermentations were carried out in mixed synthetic inhibitor cocktail with glucose and xylose (AP1, Fig. 3A).

The inclusion of the *PAD1*-*ICT1* combination in both the first and the third round of integration, however, seemed to increase the sensitivity of the yeast towards weak acids at concentrations found in the sugarcane bagasse pretreatment liquor (Table 1), as inferred from the inhibitor tolerance assays (Fig. 1C) and drop in growth in synthetic inhibitor media containing only weak acids (Fig. 2C). This could explain that the TFA7 transformant, that only contains two gene cassettes, was able to outperform CelluX^TM^1 and, in some cases, TP1 and AP1 as well. Alternatively, we speculate that this could also be linked to a lower metabolic burden as compared to TP1 and AP1 (Table 3). In terms of resistance towards cinnamic acids, there was no significant difference between the parental strain and the last set of transformants (Fig. 2A). In contrast, this resistance phenotype was evident in the first round of integration for the *PAD1*-*ICT1* combo (PI3, Fig. 2A) together with increased susceptibility to higher temperatures (Fig. 1C). The reduced thermo-tolerance could be due to an excess fluidity of the membrane caused by a higher proportion of unsaturated fatty acids incorporated by the ICT1 protein (1-acylglycerol-3-phosphate *O*-acyltransferase) [43].

The tolerance of the yeast towards lignocellulosic-derived inhibitors is also dependent on the carbon source in the fermentation media, and xylose metabolism is much more susceptible than glucose’s [2]. However, there is limited information on the possible interaction between genes conferring inhibitor tolerance, and the genes associated with the XI pathway, especially in industrial strains of *S.* *cerevisiae*. Our results demonstrate the inter-linkage between carbon metabolism and microbial inhibitor resistance. During fermentations with inhibitor cocktail media containing xylose as only carbon source, the conversion of furfural was decreased drastically in all the strains (Fig. 3B). However, this reduction was not as severe in the case of the transformants (2.4–4 times less) compared to the parental strain (7.4 times less) (Fig. 3B). Invariably, the true test of inhibitor resistance is fermentation ability exhibited with lignocellulose hydrolysates. Fermentations in SSL presented a unique challenge to the strain development as it is both xylose rich and contain microbial inhibitors unique to the paper and pulp production process such as lignosulfonates and high concentrations of Ca^+2^ or Mg^+2^ ions besides the typical compliment of weak acids, furans and phenolics (Table 1).

In 2% YPD supplemented with 40% v/v SSL, transformants TP1 and AP1 outperformed strain CelluX^TM^1 (4.8% increment on ethanol yield for API1, 11.84% increment on ethanol yield for TP1), confirming enhanced inhibitor resistance phenotypes (Table 3). Given the poor performances of strains in xylose-only fermentations supplemented with inhibitor cocktail, it was expected that strains would not be able to tolerate SSL well. However, this improvement was observed despite a reduction in the xylose consumption compared to the parental strain, especially for the API1 transformant (Table 3). However, the 80% v/v SSL YPD media proved too toxic for all the strains, but it should be noted that the strains were specifically developed with furans, weak acids and phenolic compounds in mind. Hardwood-SSL contains atypical microbial inhibitors such as MgO, lignosulfonates and SO_2_ which the industrial yeast strains are as yet unable to tolerate at such high concentrations (Table 1).

Unexpectedly, the ethanol production from xylose in the selected transformants was also reduced when fermentations were carried out with no inhibitors present (Table 3, values for YPX). Nonetheless, the transformants containing the *TAL1* + *FDH1* combination from the first integration (TFA7, TP1) were less influenced with about 10% reduction on xylose consumption compared to an almost 58% reduction in the AP1 transformant. The positive synergism of the overexpression of these two genes has been documented for a recombinant xylose-fermenting *S.* *cerevisiae* laboratory strain, i.e. the ethanol production from xylose was improved, despite the media containing both acetic acid (1.8 g L^−1^) and formic acid (0.96 g L^−1^) [47].

Different gene interactions between gene combinations with no differences in gene copy numbers were observed, suggesting there might also have been a possible “position effect” influencing the multi-inhibitor resistance phenotypes. It is tempting to speculate that the initial or first integration events exhibited a more dominant phenotype because the first cassettes integrated into highly active and assessable sites, whereas subsequent integrations were relegated to less active areas, a so-called “positional effect”. As such, the transformation efficiency was reduced with each integration cycle. This positional effect could explain the higher furfural conversion of API1 (Fig. 3A), and the interaction of *TAL1* + *FDH1* on xylose metabolism when the combination was the first integration cassette (Table 3: TFA7, TP1), as well as the increment of conversion of cinnamic acids only when the *PAD1* gene was on the first integration cassette (Fig. 2A).

Additional study into advanced strain development strategies for the manipulation of complex phenotypes, such as microbial inhibitor resistance with a minimal detrimental impact on xylose fermentation on XI engineered industrial yeasts is required. Furthermore, elucidating how gene location, gene dosage and copy number influence exhibited phenotypes, would improve the tuning of the transformation process by adjusting the DNA concentration and/or sequence of integration according to genes (function, size) [48]. Combining targeted rational engineering with techniques such as evolutionary engineering or genome shuffling may pave the way forward in the manipulation of complex phenotypes [49].

## Conclusions

The efficient conversion of sugars (glucose and xylose) in the presence of microbial inhibitors for lignocellulose-derived biofuels production still remains a challenge. This study aimed to evaluate novel gene combinations that confer resistance to multiple inhibitors (cumulative resistance phenotypes) in recombinant xylose-capable industrial yeast strains. The sequential delta-integration of these genes resulted in strains with improved tolerance towards furans and formic acid, but these acquired abilities somehow negatively influenced the xylose consumption capacity of the yeast. Nevertheless, there were combinations of genes where this impact was minimal, specifically when the sequence of integrations was *TAL1* + *FDH1* followed by *ARI1* + *ADH6* (and *PAD1* + *ICT1*). Despite the reduced xylose fermentation, selected strains could outperform the parental strain when grown on synthetic media supplemented with 40% (v/v) xylose-rich SSL hydrolysate. This study highlighted inhibitor resistance as a complex phenotype and contributes towards developing advanced strain development techniques based on positive gene interactions/mechanisms to develop “hardened” multi-inhibitor resistance xylose-capable *S.* *cerevisiae* strains.

## Materials and methods

### Microbial strains and culture conditions

*S.cerevisiae* CelluX^TM^1 (Leaf by Lesaffre, France) was selected as an industrial strain for rational yeast engineering. *S.* *cerevisiae* CelluX^TM^1 and transformants were routinely cultivated, selected and screened by using YPD (20 g L^−1^ glucose, 10 g L^−1^ yeast extract and 20 g L^−1^ peptone; Merck—Darmstadt, Germany) media supplemented with 300–400 µg mL^−1^ of the appropriate antibiotics or combination of antibiotics namely; hygromycin B (Calbiochem, San Diego, USA), geneticin (Melford laboratories, Ipswich, UK) and zeocin (Melford, Ipswich, UK). Strains were pre-cultured in synthetic complete (SC-X) media at pH 5 containing 20 g L^−1^ glucose and 20 g L^−1^ xylose, 5 g L^−1^ (NH_4_)_2_SO_4_, 1.67 g L^−1^ YNB w/o amino acids, 3 g L^−1^ KH_2_PO_4_ and 100 mM potassium phthalate, supplemented with 20% inhibitor cocktail (20%-IC) containing 0.2 g L^−1^ cinnamic acid, 0.1 g L^−1^ HMF, 1.5 g L^−1^ furfural, 1.2 g L^−1^ acetic acid and 0.16 g L^−1^ formic acid (Sigma Aldrich, St. Louis, USA). Pre-cultures were incubated at 30 °C and shaking at 200 rpm. Growth curves of select strains were conducted in YPD and YPDX (20 g L^−1^ glucose, 10 g L^−1^ yeast extract, 20 g L^−1^ peptone, 20 g L^−1^ xylose; Merck, Darmstadt, Germany) media, incubated at 30 °C, shaking at 200 rpm and sampled at 3-h intervals for 24 h. *Escherichia* *coli* DH5α (Life Technologies-CA, USA) was used for plasmid propagation and cloning. *E.coli* transformants were cultivated at 37 °C in Luria–Bertani (LB) media (1% tryptone, 0.5% yeast extract, 1% NaCl; Merck, Darmstadt, Germany) supplemented with 100 µg mL^−1^ ampicillin (Roche, Johannesburg, South Africa).

### Construction of plasmids

Standard protocols for DNA manipulation were followed [50]. Genomic DNA was extracted from *S.* *cerevisiae* BY4742∆FPS1 [51] and used as template DNA for amplification of target genes open reading frame. Target genes *ARI1*, *ADH6*, *FDH1*, *ICT1*, *PAD1*, and *TAL1* were amplified via PCR using the Phusion^®^ high-fidelity DNA polymerase (New England Biolabs, Ipswich, USA) and appropriate primers (Table 4) on an Applied Biosystems 2720 thermocycler (Life Technologies, CA, USA) according to the manufacturers’ recommendations. The primers introduced *Pac*I and *Asc*I restriction sites required for directional cloning into the delta-integration plasmids, pBZD [52], pBKD [52] and pBHD [53]. PCR products were initially ligated into the pCLoneJET 1.2 commercial vector (Thermo Scientific, Waltham, USA) according to the manufacturers’ guidelines. Gene sequences were verified using the dideoxy chain termination method and an ABI PRISM™ 3100 genetic analyser (Applied Biosystems, Waltham, USA) at Central Analytical Facility (CAF) of Stellenbosch University.

**Table 4:** Primers used in the study

Gene	Primer	Primer 5–3′ sequence	References
*ADH6*	ADH6-F	GCGCCTTAATTAAATGTCTTATCCTGAGAA	This study
*ADH6*	ADH6-R	GTTAGGCGCGCCCTAGTCTGAAAATTC	This study
*PAD1*	PAD1-F	GGCCTTAATTAAATGCTCCTATTTCCAAGAAG	This study
*PAD1*	PAD1-R	GATTGGCGCGCCTTACTTGCTTTTTATT	This study
*ICT1*	ICT1-F	GGCCTTAATTAAATGTGGACAAACACTTTCAAATGG	This study
*ICT1*	ICT1-R	GATTGGCGCGCCTTACTTTGACAGGAAC	This study
*ARI1*	ARI1-F	GGCCTTAATTAAATGACTACTGATACCACTG	This study
*ARI1*	ARI1-R	GATTGGCGCGCCTTAGGCTTCATTT	This study
*FDH1*	FDH1-F	GGCCTTAATTAAATGTCGAAGGGAAAGG	This study
*FDH1*	FDH1-R	GATCGGCGCGCCTTATTTCTTCTGT	This study
*TAL1*	TAL1-F	GCGCTTAATTAAATGTCTGAACCAGCTC	This study
*TAL1*	TAL1-R	GATAGGCGCGCCTTAAGCGGTAACTTTC	This study
*FPS1*	FPS1-L	CCGAAGCTTATGAGTAATCCTCAAAAAGC	[41]
*FPS1*	FPS1-R	CCAGAGCTCTCATGTTACCTTCTTAGCATT	[41]
*CUP1*	CUP1-L	CTTGGTACCTGGGCGCTATACGTGCATATG	[41]
*PGK1*	PGKseq-L	CTAATTCGTAGTTTTTCAAGTTCTTAGATGC	[54]
*ENO2*	BKDENOpt-L	TCAGTCTAGAGCGGCCGCCTTCTAGGCGGGTTATC	This study

Restriction sites underlined

The first gene expression cassettes were constructed by directional cloning of *ARI1*, *TAL1* or *PAD1* into plasmids pBKD1, pBHD1 or pBZD1 (D1), respectively, containing the constitutive *PGK1* gene promoter and terminator sequences. Secondary gene expression cassettes were constructed by directional cloning of *ADH6*, *FDH1* or *ICT1* into plasmid pBKD2 (D2) containing the constitutive *ENO1* gene promoter and terminator sequences. Double gene expression cassettes were generated by sub-cloning the pBKD2 ENO1_pt_ gene cassettes as a *Spe*I*/Not*I fragment into corresponding pB(K/H/Z)D1 plasmids to yield single delta plasmids with both PGK_pt_ and ENO_pt_ expression cassettes (see Additional file 1: Table S1). All plasmids used and constructed in the study are listed in Table 5.

**Table 5:** Final plasmids and yeast strains used in study

Plasmids/strain	Relevant genotype		References	
Plasmids				
pBKD1		*bla* δ*-site* *PGK1*_*P*_*-PGK1*_*T*_ *kanMX* δ*-site*		[52]
pBKD-AA		*bla* δ*-site* *PGK1*_*P*_*-ARI1-PGK1*_*T*_ *kanMX* *ENO1*_*P*_*-ADH6-ENO1*_*T*_ δ*-site*		This work
pBHD1		*bla* δ*-site* *PGK1*_*P*_*-PGK1*_*T*_ *hphNT* δ*-site*		[53]
pBHD-TF		*bla* δ*-site* *PGK1*_*P*_*-TAL1-PGK1*_*T*_ *hphNT* *ENO1*_*P*_*-FDH1-ENO1*_*T*_ δ*-site*		This work
pBZD1		*bla* δ*-site* *PGK1*_*P*_*-PGK1*_*T*_ *ShBle* δ*-site*		[52]
pBZD-PI		*bla* δ*-site* *PGK1*_*P*_*-PAD1-PGK1*_*T*_ *ShBle* *ENO1*_*P*_*-ICT1-ENO1*_*T*_ δ*-site*		This work
*S.cerevisiae* strains				
*S.cerevisiae* CelluX^TM^1		*BUD5::ADH1*_*P*_*-XKS1-CYC1*_*T*_*TAL1::PGK1*_*P*_*-TAL1-CYC1*_*T*_*TKLI::TDH3*_*P*_*-TKL1-CYC1*_*T*_*RPE1::TDH3*_*P*_*-RPE1-CYC1*_*T*_*RKI1::TDH3*_*P*_*-RKI1-CYC1*_*T*_*HO::PsXYLZ-HYGROBUD5::CpXI-BLASTGRE3/∆GRE3*		[19]
*S.cerevisiae* AA6		CelluX^TM^1 *FPS1*/∆*FPS1* *PGK1*_*P*_*-ARI1-PGK1*_*T*_ *kanMX* *ENO1*_*P*_*-ADH6-ENO1*_*T*_		This work
*S.cerevisiae* TF2		CelluX^TM^1 *FPS1*/∆*FPS1* *PGK1*_*P*_*-TAL1-PGK1*_*T*_ *hphNT* *ENO1*_*P*_*-FDH1-ENO1*_*T*_		This work
*S.cerevisiae* PI3		CelluX^TM^1 *FPS1*/∆*FPS1* *PGK1*_*P*_*-PAD1-PGK1*_*T*_ *ShBle* *ENO1*_*P*_*-ICT1-ENO1*_*T*_		This work
*S.cerevisiae* TFA7		CelluX^TM^1 *FPS1*/∆*FPS1* *PGK1*_*P*_*-TAL1-PGK1*_*T*_ *hphNT* *ENO1*_*P*_*-FDH1-ENO1*_*T*_ *PGK1*_*P*_*-ARI1-PGK1*_*T*_ *kanMX* *ENO1*_*P*_*-ADH6-ENO1*_*T*_		This work
*S.cerevisiae* TP1		TFA7 *PGK1*_*P*_*-PAD1-PGK1*_*T*_ *ShBle* *ENO1*_*P*_*-ICT1-ENO1*_*T*_		This work
*S.cerevisiae* AP1		AA6 *PGK1*_*P*_*-TAL1-PGK1*_*T*_ *hphNT* *ENO1*_*P*_*-FDH1-ENO1*_*T*_ *PGK1*_*P*_*-PAD1-PGK1*_*T*_ *ShBle* *ENO1*_*P*_*-ICT1-ENO1*_*T*_		This work

### Yeast transformation and screening

#### FPS deletion stains

The first step of the rational engineering strategy was to disrupt *FPS1* to generate *FPS1* deletion strains using plasmid pYFCUP1 [41]. Plasmid DNA was propagated and extracted using cetyltrimethylammonium bromide (CTAB) plasmid extraction protocol [50], and used as a template to amplify the *FPS1L*-*CUP1*-*FPS1R* insert by PCR using Phusion® high-fidelity DNA polymerase (New England Biolabs, Ipswich, USA) and appropriate primers. The 3030 bp linear PCR product was separated on 1% agarose gel to confirm the insert. The PCR product was then purified using GeneJet PCR purification kit (Thermo Scientific, Waltham, USA) and transformed into *S.* *cerevisiae* CelluX^TM^1 by electroporation using a Bio-Rad Gene-Pulser Apparatus (1.4 kV, 200 OHMS, and 25 µF). Transformants were incubated in 2% YPDS (20 g L^−1^ glucose, 10 g L^−1^ yeast extract, 20 g L^−1^ peptone, 1 M sorbitol) media at 30 °C for 4–5 h, plated on YPDS agar plates supplemented with 7 mM and 8 mM CuSO_4_ and incubated for 72 h at 30 °C. Successful transformants were confirmed with PCR using FPS1-L forward and CUP1-L reverse primers. Partial *FPS1* deletion was confirmed and attributed to the aneuploidy nature of parental strain. The partial *FPS1* deletion strains were screened in 70-mL fermentations using 2% YPDX supplemented with sugarcane pre-treatment liquor/hydrolysate to a concentration of 50% v/v. Fermentations were sampled at 24-h intervals for 7 days and samples were analysed for fermentations products as described in HPLC analysis sector.

#### Strain construction

The first round of delta integration involved the transformation of nine distinct double-gene expression cassettes into the partial *FPS1* deletion CelluX^TM^1 strain. All integration plasmids were digested with either *Bst*11071 or *Xho*I (Thermo Scientific—Waltham, USA) according to the manufacturer recommendations and transformed into partial *FPS1* deletion CelluX^TM^1 strain by electroporation (1.4 kV, 200 OHMS, and 25 µF) using a Bio-Rad Gene-Pulser Apparatus. Transformants were recovered on 2% YPD supplemented with appropriate antibiotics and confirmed via PCR using PGKseq-L (D1) and BKDENOpt-L (D2) as forward primers in conjunction with gene-specific reverse primers to confirm the complete double-gene insert (Table 4). The transformation frequency of the delta plasmids allowed for a range of copies to be integrated, thus preliminary plate screenings were conducted to identify transformants with higher copy numbers. The plate assays were based on antibiotic resistance, i.e. higher copy numbers transformants exhibit increased antibiotic resistance. The screening was done on 2% YPD plates supplemented with increasing concentrations of appropriate antibiotics. Ten transformants were selected and underwent high-throughput screening to determine growth and ethanol yields. Transformants were pre-cultured in 2% YPD supplemented with 20% v/v sugarcane hydrolysate for 24 h and inoculated to an optical density (OD_600_) of 1 into 5 mL media composed of 2% YPD supplemented with 65% v/v sugarcane hydrolysate. Fermentations were conducted in capped 15-mL conical tubes incubated at 30 °C, on a rotary wheel at 100 rpm, for 120 h with endpoint sampling for fermentation products and growth (OD_600_). *S.* *cerevisiae* CelluX^TM^1 was used as a parental reference strain in all screening fermentations.

The second round of delta integration involved the integration of pBKD-AA and pBHD-TF plasmids into TF2 (*TAL1* + *FDH1*) and AA6 (*ARI1* + *ADH6*) strains, respectively, thus generating strains with AA and TF double-gene expression cassettes in different combinations. Plasmids were linearized with *Xho*I (Thermo Scientific, Waltham, USA) and transformed into selected strains via electroporation. Subsequently, transformants were recovered on 2% YPD plates supplemented with appropriate antibiotics and confirmed via PCR using appropriate primer combinations. Twenty-four confirmed transformants were selected for each combination (TF2 + pBKD-AA or AA6 + pBHD-TF) and screened in 2% YPD supplemented with 65% v/v hydrolysate.

The final (third round) delta integration involved the addition of the pBZD-PI expression cassette to the second round of gene combinations to give TF2-pBKD-AA + pBZD-PI and AA6-pBHD-TF + pBZD-PI overexpression strains. Transformation efficiency decreased after each round of sequential integration, with only six strains per combination recovered and confirmed via PCR after the third round of integration. Strains were screened in 2% SC-X supplemented with 65% v/v hydrolysate and spiked with 20 g L^−1^ furfural and formic acid and final strains selected overexpressed six inhibitor resistance genes in different combinations. Strain names were derived from the initials of the genes inserted (e.g. TF is *TAL1* + *FDH1*). For the second-round transformants, the initial of the second integration (D1 gene) was added to initials of the first integration (e.g. TF + *ARI1* resulting in TFA), whereas, for the third-round transformants, the initial of the first integration D1 gene and initial of the third D1 gene were combined (e.g. *TAL1* (D1 gene first integration) + *PAD1* (D1 gene third integration) = TP). The copy number of the introduced genes was determined using qPCR (service provided by Inqaba Biotechnical Industries (Pty) Ltd, South Africa). The *ALG9* gene was used as housekeeping gene.

### Enzymatic activity assays

Different enzyme assays were conducted to determine the enzymatic detoxification capacity of transformants as compared to the parental CelluX^TM^1 strain. Transformants overexpressing *PAD1*, *ARI1*, *ADH6* and *FDH1* with direct enzymatic detoxification mechanisms were analysed in vivo for detoxification activity using crude cell extract. Strains were grown on appropriate antibiotic selective plates for 48 h. Single colonies were suspended in 1 mL SC media to an OD_600_ of 1 (~ 0.6 g L^−1^ DW). Cells were harvested via centrifugation and washed first with PBS buffer (8.01 g L^−1^ NaCl, 0.2 g L^−1^ KCl, 1.78 g L^−1^ Na_2_HPO_4_.H_2_O, 0.27 g L^−1^ KH_2_PO_4_) followed by two washing steps in lysis buffer (10 mM phosphate buffer, 2 mM EDTA, 1 mM PMSF, pH 7). After the second wash, 100 µL glass beads (0.4 mm diameter, Sigma Aldrich, St. Louis USA) were added to cell pellets with 100 µL lysis buffer in 2-mL Eppendorf tubes and chilled on ice for 2 min. Cells were disrupted in ten cycles with each cycle consisting of 1 min of vigorous vortexing followed by 1-min cooling steps on ice. The cell extract was centrifuged at 13,000 rpm for 5 min at 4 °C and supernatant was aspirated and analysed for enzymatic activity. Aldehyde reductase activity was assayed according to Petersson et al. [37] with modifications. The reaction mixture consisted of 10 mM furfural substrate and 100 µM NADPH cofactor in 100 mM potassium phosphate buffer pH 7.0. The reaction was started with the addition of 20 µL crude cell extract and assays were incubated at 30 °C for 45 min. Formate dehydrogenase (FDH) activity was monitored according to Hasunuma et al. [30] with modifications. Reaction mixture consisted of 50 mM sodium formate substrate and 0.4 mM NAD + cofactor in 50 mM potassium phosphate buffer pH 7.0. FDH reaction was started with the addition of 20 µL crude cell extract and assays were incubated at 30 °C for 45 min. Phenolic detoxification was determined according to Richard et al. [33] with adjustments. The reaction mixture consisted of 0.4 mM cinnamic acid substrate suspended in 20 mM sodium phosphate buffer pH 7.0. The reaction was initiated with the addition of 20 µL crude cell extract and incubated at 30 °C for 45 min. All assays were deactivated via acidification with the addition of 10% v/v H_2_SO_4_ and stored at − 20 °C until analysis.

### Inhibitor tolerance assays

The inhibitor tolerance phenotypes of transformants were assessed using tolerance plate assays to determine synergistic/antagonistic dynamics in strains containing the different gene expression cassettes. Transformants were streaked out on 2% YPD plates supplemented with appropriate antibiotics and incubated at 30 °C for 48 h. Single colonies of strains were suspended into 200 µL 2% SC and suspensions were made to an OD_600_ of 5, 1, 0.5, 0.1 and 0.01. Inhibitor tolerance plates consisted of 2% SC agar supplemented with either 0.6 g L^−1^ syringaldehyde, 1 g L^−1^ furfural, or 6 g L^−1^ acetic acid and 0.8 g L^−1^ formic acid. *S.* *cerevisiae* CelluX^TM^1 was used as parental reference strain. The AA6, TF2 and PI3 strains are inhibitor specific for furfural, weak acids and phenolics, respectively, and were used as references for the phenotype of the first double-gene expression cassettes. Transformants were spotted onto inhibitor tolerance plates (5 µL) and incubated at 30 °C for 48–72 h. Strains were also cultivated on 2% SC plates and subjected to a higher temperature of 37 °C as this temperature is more industrially relevant.

### Fermentations with single inhibitor group and inhibitor cocktail

Fermentations were conducted in 2% SC-X minimal media supplemented with either; 1 g L^−1^ cinnamic acid at pH 5.0, 5 g L^−1^ furfural and 0.5 g L^−1^ HMF at pH 5.0, or 5 g L^−1^ acetic acid and 0.81 g L^−1^ formic acid at pH 5.0. Fermentations in 2% SC-X supplemented with inhibitor cocktail (IC) contained 6 g L^−1^ acetic acid, 0.8 g L^−1^ formic acid, 5 g L^−1^ furfural, 0.5 g L^−1^ HMF and 0.5 g L^−1^ cinnamic acid. The concentration of cinnamic acid at 0.5 g L^−1^ was selected to account for the total phenolic content of spent sulphite liquor (SSL). Strains were pre-cultured in 50 mL 20%-IC SC-X media pH 5.0 until late exponential/early stationary phase and inoculated into 50 mL 2% SC-X or 2% SCX (xylose only) media supplemented with appropriate inhibitors, to an OD_600_ of 1. Fermentations were incubated at 30 °C, shaking at 200 rpm for 120 h, with sampling at 24-h intervals. Fermentations were inoculated in triplicate with *S.* *cerevisiae* CelluX^TM^1 as an industrial and parental reference strain. Media controls were included in all fermentations to account for evaporation of volatile inhibitor compounds. The inhibitor cocktail composition is based in part on inhibitor concentrations found in both SSL and sugarcane steam explosion liquor. Synthetic media supplemented with inhibitors were selected to exercise better control over experimental parameters given the inherent unknown/unquantifiable mix of inhibitors present in hydrolysate/SSL.

### Lignocellulose hydrolysate and SSL fermentations

Fermentations with concentrated SSL was conducted to ascertain strain performance under industrially relevant fermentation conditions. SSL was kindly provided by Sappi Saiccor (Umkomaas, South Africa) which uses an acid-based sulphite pulping process. Strains were pre-cultured in 2% SC-X media supplemented with 20% v/v SSL to late exponential/early stationary phase (OD_600_ > 10) and inoculated into 100-mL serum bottles with 50 mL fermentation media to an OD_600_ of 1 (~ 0.6 g L^−1^ DW). Fermentation media consisted of 2% SC media (20 g L^−1^ glucose, 5 g L^−1^ (NH_4_)_2_SO_4_, 1.67 g L^−1^ YNB w/o aa, 3 g L^−1^ KH_2_PO_4_ and 100 mM potassium phthalate), supplemented with either 40% (pH 5) or 80% (pH 4.5) v/v concentrated SSL. Samples were taken every 24 h and analysed via HPLC. Sugarcane hydrolysate was generated via steam pretreatment and pressing of pre-treated material. First, sugarcane bagasse was water impregnated for 24 h and dewatered using a spin dryer. The steam explosion pre-treatment experiment was performed at 205 °C at a residence time of 13.5 min. The slurry was then pressed and the hydrolysate was collected, aliquot and frozen for storage at − 20 °C until use.

### Chemical composition and analytical analysis

High-performance liquid chromatography (HPLC) was conducted to determine glucose, xylose, ethanol, glycerol, acetic acid and formic acid concentrations. Samples were run on an Aminex HPX-87H Column equipped with a Cation-H Micro-Guard Cartridge (Bio-Rad, Johannesburg, South Africa) at column temperature of 65 °C with a mobile phase of 5 mM sulphuric acid and a flow rate of 0.6 ml min^−1^. Peaks were detected with an RI detector (Shodex, RI-101) operated at 45 °C. Furfural and HMF were analysed on a Luna C18 (2) reversed-phase column equipped with a Luna C18 (2) precolumn (Phenomenex). Mobile phases used for elution was 5 mM trifluoroacetic acid in water (phase A) and 5 mM trifluoroacetic acid in acetonitrile (phase B), the column temperature was set to 25 °C and the flow rate at 0.7 ml min^−1^. Separation occurred via gradient elution, 5% mobile phase B, increasing to 11% phase B over 14 min followed by an increase to 40% phase B over 3 min and was then kept constant at 40% for 2 min. This was followed by a decrease to 5% phase B over 5 min and ended in a final step of constant composition at 5% B for 4 min to equilibrate. Peaks were detected with a Dionex Ultimate 3000 diode array detector at 215 nm and 285 nm. Phenolic compounds were analysed with Chromeleon 6.8 software on a Dionex 3000 System with UV detector at 285 nm equipped with a Waters XSelect C18 Column (4.6 × 250 mm). Mobile phases used for elution was water (phase A) and acetonitrile (phase B) at a flow rate of 0.7 mL min^−1^ [55]. The SO_2_ content was measured via the Ripper titration [56] at the Department of Wine Biotechnology, whereas magnesium oxide (MgO) content via ICP-MS, at the Department of Geology, Stellenbosch University.

### Calculations and statistical analysis

All experiments were conducted in triplicate. Data were analysed using Microsoft Excel data analysis tools, whereby triplicate values were subjected to analysis of variance (ANOVA) and *p* < 0.05 was considered significant for this study. Ethanol yields (*Y*_E/TS_) were calculated as final ethanol (g L^−1^) divided by total sugar (g L^−1^). Ethanol productivity was calculated as ethanol concentration (g L^−1^) divided by fermentation time (h). The metabolic yield of ethanol was calculated as final ethanol concentration (g L^−1^) divided by total consumed sugar (g L^−1^) throughout the fermentation, compared to the theoretical maximum metabolic yield of 0.51 (g g^−1^) expressed as a fraction (%). The growth rate as µmax was determined during the exponential growth phase by plotting the natural logarithm values as a function of time.

## Supplementary Information

**Additional file 1**: Gene target improvements to transformants in literature and in the current study. The gene products, functions and reported strain improvements attributed to overexpression of various gene targets or the deletion of the *FPS1* gene as found in the literature (Table S1), the fermentation parameters and % inhibitor conversion of partial *FPS1* deletion transformants in 2% SC-X media supplemented with 65% v/v sugarcane hydrolysate at 168 h (Table S2). The statistical analysis of partial *FPS1* transformants using ANOVA and T-test, with p < 0.05 as statistically significant were also included (Table S3). The data for the gene copy numbers determined via qPCR are included (Table S4). Also included are the in vivo detoxification phenotypes exhibited by the final TFA7, AP1 and TP1 transformants as mg L^−1^ h in various detoxification enzyme assays (Table S5).

**Additional file 2**: Growth and ethanol yields in round 1 and round 2 transformants. The % increment in growth and ethanol yield relative to parental strain for the 1st round transformants (Figure S1) and 2nd round transformants (Figure S2) in fermentations with 2% SC-X supplemented with 65% v/v sugarcane hydrolysate at 120 h.

## Data Availability

All data generated or analysed during this study are included in this published article [and its Additional files]. The datasets used and/or analysed during this study are available from the corresponding author on reasonable request.

## References

[1] Jansen MLA, Bracher JM, Papapetridis I, Verhoeven MD, de Bruijn H, de Waal PP (2017). *Saccharomyces**cerevisiae* strains for second-generation ethanol production: from academic exploration to industrial implementation. FEMS Yeast Res.

[2] Deparis Q, Claes A, Foulquié-Moreno MR, Thevelein JM (2017). Engineering tolerance to industrially relevant stress factors in yeast cell factories. FEMS Yeast Res.

[3] Kumar B, Bhardwaj N, Agrawal K, Chaturvedi V, Verma P (2020). Current perspective on pretreatment technologies using lignocellulosic biomass: an emerging biorefinery concept. Fuel Process Technol.

[4] Bhatia SK, Jagtap SS, Bedekar AA, Bhatia RK, Patel AK, Pant D (2020). Recent developments in pretreatment technologies on lignocellulosic biomass: effect of key parameters, technological improvements, and challenges. Bioresour Technol.

[5] Jönsson LJ, Martín C (2016). Pretreatment of lignocellulose: formation of inhibitory by-products and strategies for minimizing their effects. Bioresour Technol.

[6] Zhao X, Zhang L, Liu D (2012). Biomass recalcitrance. Part I: the chemical compositions and physical structures affecting the enzymatic hydrolysis of lignocellulose. Biofuel Bioprod Bior..

[7] Pienkos PT, Zhang M (2009). Role of pretreatment and conditioning processes on toxicity of lignocellulosic biomass hydrolysates. Cellulose.

[8] Klinke HB, Olsson L, Thomsen AB, Ahring BK (2003). Potential inhibitors from wet oxidation of wheat straw and their effect on ethanol production of *Saccharomyces**cerevisiae*: wet oxidation and fermentation by yeast. Biotechnol Bioeng.

[9] Cunha JT, Romaní A, Costa CE, Sá-Correia I, Domingues L (2019). Molecular and physiological basis of *Saccharomyces**cerevisiae* tolerance to adverse lignocellulose-based process conditions. Appl Microbiol Biotechnol.

[10] Brandt BA, Jansen T, Görgens JF, Zyl WH (2019). Overcoming lignocellulose-derived microbial inhibitors: advancing the *Saccharomyces**cerevisiae* resistance toolbox. Biofuel Bioprod Bior.

[11] Branco RHR, Serafim LS, Xavier AMRB (2019). Second generation bioethanol production: on the use of pulp and paper industry wastes as feedstock. Fermentation.

[12] Kricka W, Fitzpatrick J, Bond U (2015). Challenges for the production of bioethanol from biomass using recombinant yeasts. Adv Appl Microbiol.

[13] Wahlbom CF, van Zyl WH, Jönsson LJ, Hahn-Hägerdal B, Otero RRC (2003). Generation of the improved recombinant xylose-utilizing *Saccharomyces**cerevisiae* TMB 3400 by random mutagenesis and physiological comparison with *Pichia**stipitis* CBS 6054. FEMS Yeast Res.

[14] Cunha JT, Soares PO, Romaní A, Thevelein JM, Domingues L (2019). Xylose fermentation efficiency of industrial *Saccharomyces**cerevisiae* yeast with separate or combined xylose reductase/xylitol dehydrogenase and xylose isomerase pathways. Biotechnol Biofuels.

[15] Jeffries TW (2006). Engineering yeasts for xylose metabolism. Curr Opin Biotechnol.

[16] Almeida JRM, Runquist D, Sànchez Nogué V, Lidén G, Gorwa-Grauslund MF (2011). Stress-related challenges in pentose fermentation to ethanol by the yeast *Saccharomyces**cerevisiae*. Biotechnol J.

[17] de Vilela LF, de Araujo VPG, de Paredes RS, Bon da EPS, Torres FAG, Neves BC (2015). Enhanced xylose fermentation and ethanol production by engineered *Saccharomyces**cerevisiae* strain. AMB Express.

[18] Demeke MM, Dietz H, Li Y, Foulquié-Moreno MR, Mutturi S, Deprez S (2013). Development of a D-xylose fermenting and inhibitor tolerant industrial *Saccharomyces**cerevisiae* strain with high performance in lignocellulose hydrolysates using metabolic and evolutionary engineering. Biotechnol Biofuels.

[19] Desfougeres T, Pignede G, Techel J, inventors; LESAFFRE et COMPAGINE, assignee. Pentose-fermenting strain with optimized propagation. United States patent application US 10,273,447. 2019.

[20] Feng Q, Liu ZL, Weber SA, Li S (2018). Signature pathway expression of xylose utilization in the genetically engineered industrial yeast *Saccharomyces**cerevisiae*. PLoS ONE.

[21] Zhao X, Xiong L, Zhang M, Bai F (2016). Towards efficient bioethanol production from agricultural and forestry residues: exploration of unique natural microorganisms in combination with advanced strain engineering. Bioresour Technol.

[22] Caspeta L, Castillo T, Nielsen J (2015). Modifying yeast tolerance to inhibitory conditions of ethanol production processes. Front Bioeng Biotechnol.

[23] Almeida JR, Bertilsson M, Gorwa-Grauslund MF, Gorsich S, Lidén G (2009). Metabolic effects of furaldehydes and impacts on biotechnological processes. Appl Microbiol Biotechnol.

[24] Ma M, Liu ZL (2010). Comparative transcriptome profiling analyses during the lag phase uncover *YAP1*, *PDR1*, *PDR3*, *RPN4*, and *HSF1* as key regulatory genes in genomic adaptation to the lignocellulose derived inhibitor HMF for *Saccharomyces**cerevisiae*. BMC Genomics.

[25] Pandey AK, Kumar M, Kumari S, Kumari P, Yusuf F, Jakeer S (2019). Evaluation of divergent yeast genera for fermentation-associated stresses and identification of a robust sugarcane distillery waste isolate *Saccharomyces**cerevisiae* NGY10 for lignocellulosic ethanol production in SHF and SSF. Biotechnol Biofuels.

[26] Fletcher E, Gao K, Mercurio K, Ali M, Baetz K (2019). Yeast chemogenomic screen identifies distinct metabolic pathways required to tolerate exposure to phenolic fermentation inhibitors ferulic acid, 4-hydroxybenzoic acid and coniferyl aldehyde. Metab Eng Elsevier.

[27] De Witt RN, Kroukamp H, Volschenk H (2019). Proteome response of two natural strains of *Saccharomyces**cerevisiae* with divergent lignocellulosic inhibitor stress tolerance. FEMS Yeast Res.

[28] Chen Y, Sheng J, Jiang T, Stevens J, Feng X, Wei N (2016). Transcriptional profiling reveals molecular basis and novel genetic targets for improved resistance to multiple fermentation inhibitors in *Saccharomyces**cerevisiae*. Biotechnol Biofuels.

[29] Zhang MM, Xiong L, Tang YJ, Mehmood MA, Zhao ZK, Bai FW (2019). Enhanced acetic acid stress tolerance and ethanol production in *Saccharomyces**cerevisiae* by modulating expression of the de novo purine biosynthesis genes. Biotechnol Biofuels.

[30] Hasunuma T, Sung KM, Sanda T, Yoshimura K, Matsuda F, Kondo A (2011). Efficient fermentation of xylose to ethanol at high formic acid concentrations by metabolically engineered *Saccharomyces**cerevisiae*. Appl Microbiol Biotechnol.

[31] Li YC, Gou ZX, Liu ZS, Tang YQ, Akamatsu T, Kida K (2014). Synergistic effects of *TAL1* over-expression and *PHO13* deletion on the weak acid inhibition of xylose fermentation by industrial *Saccharomyces**cerevisiae* strain. Biotechnol Lett.

[32] Hasunuma T, Ismail KSK, Nambu Y, Kondo A (2014). Co-expression of *TAL1* and *ADH1* in recombinant xylose-fermenting *Saccharomyces**cerevisiae* improves ethanol production from lignocellulosic hydrolysates in the presence of furfural. J Biosci Bioeng.

[33] Richard P, Viljanen K, Penttilä M (2015). Overexpression of *PAD1* and *FDC1* results in significant cinnamic acid decarboxylase activity in *Saccharomyces**cerevisiae*. AMB Express.

[34] Mukai N, Masaki K, Fujii T, Kawamukai M, Iefuji H (2010). *PAD1* and *FDC1* are essential for the decarboxylation of phenylacrylic acids in *Saccharomyces**cerevisiae*. J Biosci Bioeng.

[35] Mira NP, Becker JD, Sá-Correia I (2010). Genomic expression program involving the Haa1p-Regulon in *Saccharomyces**cerevisiae* response to acetic acid. Omi A J Integr Biol.

[36] Sehnem NT, da Silva MA, Leite FCB, de Barros PW, de Morais MA, Ayub MAZ (2013). 5-Hydroxymethylfurfural induces *ADH7* and *ARI1* expression in tolerant industrial *Saccharomyces**cerevisiae* strain P6H9 during bioethanol production. Bioresour Technol.

[37] Petersson A, Almeida JRM, Modig T, Karhumaa K, Hahn-Hägerdal B, Gorwa-Grauslund MF (2006). A 5-hydroxymethyl furfural reducing enzyme encoded by the *Saccharomyces**cerevisiae**ADH6* gene conveys HMF tolerance. Yeast.

[38] Jordan DB, Braker JD, Bowman MJ, Vermillion KE, Moon J, Liu ZL (2011). Kinetic mechanism of an aldehyde reductase of *Saccharomyces**cerevisiae* that relieves toxicity of furfural and 5-hydroxymethylfurfural. Biochim Biophys Acta Proteins Proteomics.

[39] Larsson S, Nilvebrant NO, Jonsson LJ (2001). Effect of overexpression of *Saccharomyces**cerevisiae* Pad1p on the resistance to phenylacrylic acids and lignocellulose hydrolysates under aerobic and oxygen-limited conditions. Appl Microbiol Biotechnol.

[40] Liu ZL, Moon J (2009). A novel NADPH-dependent aldehyde reductase gene from *Saccharomyces**cerevisiae* NRRL Y-12632 involved in the detoxification of aldehyde inhibitors derived from lignocellulosic biomass conversion. Gene.

[41] Zhang J-G, Liu X-Y, He X-P, Guo X-N, Lu Y, Zhang B (2011). Improvement of acetic acid tolerance and fermentation performance of *Saccharomyces**cerevisiae* by disruption of the *FPS1* aquaglyceroporin gene. Biotechnol Lett.

[42] Wei N, Xu H, Kim SR, Jin YS (2013). Deletion of *FPS1*, encoding aquaglyceroporin Fps1p, improves xylose fermentation by engineered *saccharomyces**cerevisiae*. Appl Environ Microbiol.

[43] Ghosh AK, Ramakrishnan G, Rajasekharan R (2008). YLR099C (*ICT1*) encodes a soluble Acyl-CoA-dependent lysophosphatidic acid acyltransferase responsible for enhanced phospholipid synthesis on organic solvent stress in *Saccharomyces**cerevisiae*. J Biol Chem.

[44] Qi Y, Liu H, Chen X, Liu L (2019). Engineering microbial membranes to increase stress tolerance of industrial strains. Metab Eng.

[45] Narayanan V, Nogué VSI, van Niel EW, Gorwa-Grauslund MF (2016). Adaptation to low pH and lignocellulosic inhibitors resulting in ethanolic fermentation and growth of *Saccharomyces**cerevisiae*. AMB Express.

[46] Divate NR, Chen GH, Divate RD, Ou BR, Chung YC (2017). Metabolic engineering of *Saccharomyces**cerevisiae* for improvement in stresses tolerance. Bioengineered.

[47] Sanda T, Hasunuma T, Matsuda F, Kondo A (2011). Repeated-batch fermentation of lignocellulosic hydrolysate to ethanol using a hybrid *Saccharomyces**cerevisiae* strain metabolically engineered for tolerance to acetic and formic acids. Bioresour Technol.

[48] Shi S, Liang Y, Zhang MM, Ang EL, Zhao H (2016). A highly efficient single-step, markerless strategy for multi-copy chromosomal integration of large biochemical pathways in *Saccharomyces**cerevisiae*. Metab Eng.

[49] Hasunuma T, Ishii J, Kondo A (2015). Rational design and evolutional fine tuning of *Saccharomyces**cerevisiae* for biomass breakdown. Curr Opin Chem Biol.

[50] Sambrook J, Russell D. Molecular cloning: a laboratory manual. In: Joseph S, Russell DW, editors. Q Rev Biol. 2001;76:348–9.

[51] Lõoke M, Kristjuhan K, Kristjuhan A (2011). Extraction of genomic DNA from yeasts for PCR-based applications. Biotechniques.

[52] McBride JE, Delault KM, Lynd LR, Pronk JT, inventors; Dartmouth College, assignee. Recombinant yeast strains expressing tethered cellulase enzymes. United States patent application US 2010/0075363. 2010.

[53] Kroukamp H, Den Haan R, Van Wyk N, Van Zyl WH (2013). Overexpression of native *PSE1* and *SOD1* in *Saccharomyces**cerevisiae* improved heterologous cellulase secretion. Appl Energy.

[54] Van den Elzen A. Study of the termination factor like Dom34-Hbs1 complex: functional analysis of their roles in RNA quality control and in stimulating translation by dissociating inactive ribosomes. Université de Strasbourg; 2013.

[55] Petersen AM, Haigh K, Görgens JF (2014). Techno-economics of integrating bioethanol production from spent sulfite liquor for reduction of greenhouse gas emissions from sulfite pulping mills. Biotechnol Biofuels.

[56] Iland P. Techniques for chemical analysis and quality monitoring during winemaking. Patrick Iland Wine Promotions; 2000.

