# Supplementary material for: Rational engineering of Saccharomycescerevisiae towards improved tolerance to multiple inhibitors in lignocellulose fermentations
Source: Biotechnol Biofuels. 2021 Aug 28;14:173. doi: 10.1186/s13068-021-02021-w (PMC8403374; doi:10.1186/s13068-021-02021-w)
Supplement: Supplementary file 1 — Additional file 1: Gene target improvements to transformants in literature and in the current study. The gene products, functions and reported strain improvements attributed to overexpression of various gene targets or the deletion of the FPS1 gene as found in the literature (Table S1), the fermentation parameters and % inhibitor conversion of partial FPS1 deletion transformants in 2% SC-X media supplemented with 65% v/v sugarcane hydrolysate at 168 h (Table S2). The statistical analysis of partial FPS1 transformants using ANOVA and T-test, with p < 0.05 as statistically significant were also included (Table S3). The data for the gene copy numbers determined via qPCR are included (Table S4). Also included are the in vivo detoxification phenotypes exhibited by the final TFA7, AP1 and TP1 transformants as mg L−1 h in various detoxification enzyme assays (Table S5). [file 13068_2021_2021_MOESM1_ESM.docx]

**Rational engineering of *Saccharomyces cerevisiae* towards improved tolerance to multiple inhibitors in lignocellulose fermentations**

Bianca A. Brandt; Maria D.P. García-Aparicio, Johann F. Görgens; Willem H. van Zyl

# **Additional file 1: Supplementary Tables**

**Table S1**: Gene products, functions and reported strain improvements attributed to overexpression or deletion as per FPS1.

| **Gene** | **Product** | **Function** | **Deletion** | | | |
| --- | --- | --- | --- | --- | --- | --- |
| FPS1 | Aquaglyceroporin, plasma membrane channel | Involved in efflux of glycerol and xylitol, and in uptake of acetic acid, arsenite, and antimonite;  Key factor in maintaining redox balance by mediating passive diffusion of glycerol.  Aquaporin activity is required for cell survival under more harsh conditions (osmotic stress) (1) | Deletion improves xylose fermentation | | | |
| **Genes D1** | **Product** | **Function (uniport.org)** | **Overexpression** | **Integration** | | |
| ARI1 | NADPH-dependent aldehyde reductase | Reduction capabilities toward at least 14 aldehydes including common lignocellulose-derived inhibitors such as furfural, HMF, vanillin, and cinnamaldehyde (2). | Improved detoxification of 2-furaldehyde and 5-hydroxymethyl-2-furaldehyde while improving cell viability.  Enhanced ethanol tolerance (3) |  |  |  |
| TAL1 | Transaldolase, enzyme in the non-oxidative pentose phosphate pathway | Balance of metabolites in the pentose-phosphate pathway. Overexpression of *TAL1* increases the flux from the pentose phosphate pathway into the glycolytic pathway | Involved in ethanol production from xylose in the  presence of acetate and formate, furfural (4,5) |  |  |  |
| PAD1 | Flavin phenyltransferase | It has been shown that this enzyme synthesizes the essential cofactor for the associated ferulic acid decarboxylase FDC1, for decarboxilation of cinnamic acids. | Increased tolerance towards cinnamic acid, up to 0.6 mM |  |  |  |
| **Genes D2** | **Product** | **Function** | **Overexpression** | **1ST** | **2ND** | **3RD** |
| ADH6 | NADP-dependent alcohol dehydrogenase 6 | NADP-dependent alcohol dehydrogenase with a **broad** substrate specificity: HMF-reducing enzymes | - Improved growth and fermentation rate in HMF  containing media and in non-detoxified lignocellulosic  hydrolysate  -Xylose consumption rate increased and glycerol yield  decreased  (6,7) | **AA**  TA  PA | ATF | AP |
| FDH1 | Formate dehydrogenase 1 | Detoxification of exogenous formate in non-methylotrophic organisms by oxidation of formate to carbon dioxide | -Improved fermentation performance in presence of high concentration of formic acid. | AF  **TF**  PF | TFA | TP |
| ICT1 | 1-acylglycerol-3-phosphate O-acyltransferase | Involved in membrane remodeling leading to increased organic solvent tolerance. Involved in resistance to azoles and copper.  Related to the adaptation to 5-hydroxymethylfurfural  during  the lag phase (8) | Increase in phosphatidic acid and other phospholipids synthesis (mbn repair) on organic solvent exposure (9), | AI  TI  PI | - | - |

References:

1. Sabir F, Loureiro-Dias MC, Soveral G, Prista C. Functional relevance of water and glycerol channels in *Saccharomyces* *cerevisiae*. FEMS Microbiol lett. 2017;364:9:fnx080.
2. Liu ZL. Molecular mechanisms of yeast tolerance and in situ detoxification of lignocellulose hydrolysates. Appl Microbiol Biotechnol. 2011;90:3:809-25.
3. Divate NR, Chen GH, Divate RD, Ou BR, Chung YC. Metabolic engineering of *Saccharomyces* *cerevisiae* for improvement in stresses tolerance. Bioengineered. 2017;8:5:524-35.
4. Hasunuma T, Sanda T, Yamada R, Yoshimura K, Ishii J, Kondo A. Metabolic pathway engineering based on metabolomics confers acetic and formic acid tolerance to a recombinant xylose-fermenting strain of *Saccharomyces* *cerevisiae*. Microbial Cell Factories. 2011;10:1:2.
5. Hasunuma T, Ismail KS, Nambu Y, Kondo A. Co-expression of *TAL1* and *ADH1* in recombinant xylose-fermenting *Saccharomyces* *cerevisiae* improves ethanol production from lignocellulosic hydrolysates in the presence of furfural. Journal of bioscience and bioengineering. 2014;117:2:165-9.
6. Almeida JR, Röder A, Modig T, Laadan B, Lidén G, Gorwa-Grauslund MF. NADH-vs NADPH-coupled reduction of 5-hydroxymethyl furfural (HMF) and its implications on product distribution in *Saccharomyces* *cerevisiae*. Appl Microbiol Biotechnol. 2008;78:6:939-45.
7. Almeida JR, Bertilsson M, Gorwa-Grauslund MF, Gorsich S, Lidén G. Metabolic effects of furaldehydes and impacts on biotechnological processes. Appl Microbiol Biotechnol. 2009;82:4:625.
8. Ma M, Liu ZL. Comparative transcriptome profiling analyses during the lag phase uncover *YAP1*, *PDR1*, *PDR3*, *RPN4*, and *HSF1* as key regulatory genes in genomic adaptation to the lignocellulose derived inhibitor HMF for *Saccharomyces* *cerevisiae*. BMC genomics. 2010;11:1:660.
9. Ghosh AK, Ramakrishnan G, Rajasekharan R. YLR099C (*ICT1*) encodes a soluble Acyl-CoA-dependent lysophosphatidic acid acyltransferase responsible for enhanced phospholipid synthesis on organic solvent stress in *Saccharomyces* *cerevisiae*. Journal of Biological Chemistry. 2008;283:15:9768-75.

**Table S2:** Fermentation parameters and % inhibitor conversion of partial FPS1 deletion transformants.

| **Strain** | **Glucose utilization (%)** | **Xylose utilization (%)** | **Ethanol _t=168_** | | **% Inhibitor conversion vs CelluX^TM^1** | | |
| --- | --- | --- | --- | --- | --- | --- | --- |
|  |  |  | g L^-1^ | Y _P/S_ | Furans | Weak acids | Phenolics |
| CelluX^TM^1 | 100% | 49.8 | 15.2 ± 0.29 | 0.39 | n.d. | 0 | n.d. |
| C1 | 100% | 45.2 | 15.0 ± 0.33 | 0.41 | n.d. | n.d | n.d. |
| C2 | 100% | 54.6 | 15.7 ± 0.29 | 0.40 | n.d. | n.d. | n.d. |
| C3 | 100% | 50.9 | 15.4 ± 0.48 | 0.39 | n.d. | n.d. | n.d. |
| C4 | 100% | 47.7 | 15.0 ± 0.20 | 0.41 | n.d. | n.d. | n.d. |
| C5 | 100% | 53.6 | 15.5 ± 0.17 | 0.39 | n.d. | 19.8* | n.d. |
| C6 | 100% | 50.8 | 15.2 ± 0.25 | 0.39 | n.d. | 3.96* | n.d. |
| C7 | 100% | 48.1 | 15.2 ± 0.12 | 0.36 | n.d. | 13.1* | n.d. |
| C8 | 100% | 42.5 | 14.1 ± 0.58 | 0.39 | n.d. | 13.4* | n.d. |

* Formic acid only

**Table S3:** Statistical analysis of partial FPS1 transformants

| **Anova: Single Factor** | | | | | | | | |
| --- | --- | --- | --- | --- | --- | --- | --- | --- |
| SUMMARY | | |  |  |  | |  |  |
| *Groups* | *Count* | | *Sum* | *Average* | *Variance* | |  |  |
| CelluX^TM^1 | 3 | | 1.177635 | 0.392545 | 2.21E-05 | |  |  |
| C1 | 3 | | 1.167779 | 0.38926 | 0.000145 | |  |  |
| C2 | 3 | | 1.221859 | 0.407286 | 6.61E-05 | |  |  |
| C3 | 3 | | 1.205894 | 0.401965 | 0.000217 | |  |  |
| C4 | 3 | | 1.164431 | 0.388144 | 7.88E-07 | |  |  |
| C5 | 3 | | 1.231807 | 0.410602 | 0.000148 | |  |  |
| C6 | 3 | | 1.167497 | 0.389166 | 4.77E-05 | |  |  |
| C7 | 3 | | 1.159901 | 0.386634 | 3.46E-05 | |  |  |
| C8 | 3 | | 1.088351 | 0.362784 | 0.000162 | |  |  |
|  | | | | | | | | |
| ANOVA |  | |  |  |  | |  |  |
| *Source of Variation* | *SS* | | *df* | *MS* | *F* | | *P-value* | *F crit* |
| Between Groups | 0.004776 | | 8 | 0.000597 | 6.37396 | | 0.000554 | 2.510158 |
| Within Groups | 0.001686 | | 18 | 9.37E-05 |  | |  |  |
|  |  | |  |  |  | |  |  |
| Total | 0.006462 | | 26 |  |  | |  |  |
|  |  | |  |  |  | |  |  |
| **t-Test: Paired Two Sample for Means** | | | | | | | | |
|  | | *CelluX^TM^1* | | | | *C5* | | |
| Mean | | 0.392545 | | | | 0.410602 | | |
| Variance | | 2.21E-05 | | | | 0.000148 | | |
| Observations | | 3 | | | | 3 | | |
| Pearson Correlation | | 0.74231 | | | |  | | |
| Hypothesized Mean Difference | | 0 | | | |  | | |
| df | | 2 | | | |  | | |
| t Stat | | -3.39238 | | | |  | | |
| P(T<=t) one-tail | | 0.038496 | | | |  | | |
| t Critical one-tail | | 2.919986 | | | |  | | |
| P(T<=t) two-tail | | 0.076993 | | | |  | | |
| t Critical two-tail | | 4.302653 | | | |  | | |

**Table S4: Gene copy numbers using antibiotic markers geneticin (GEN), hygromycin (HYG) and Zeocin (ZEO)**

| **Target** | **Strains** | **Markers Cq** | **Avg. marker Cq** | **Absolute marker Copies** | **Copies per genome** |
| --- | --- | --- | --- | --- | --- |
| GEN | TP1 (1ng) | 19,49 |  |  |  |
| GEN | TP1 (1ng) | 19,49 | 19,51 | 41648,23 | 0,52013248 |
| GEN | TP1 (1ng) | 19,55 |  |  |  |
| GEN | AP1 (1ng) | 18,82 |  |  |  |
| GEN | AP1 (1ng) | 18,57 | 18,61 | 75509,56 | 0,971526554 |
| GEN | AP1 (1ng) | 18,45 |  |  |  |
| GEN | TFA7 (1ng) | 19,29 |  |  |  |
| GEN | TFA7 (1ng) | 19,41 | 19,37 | 45702,75 | 0,810398399 |
| GEN | TFA7 (1ng) | 19,41 |  |  |  |
| HYG | TP1 (1ng) | 20,51 |  |  |  |
| HYG | TP1 (1ng) | 20,61 | 20,57 | 41559,15 | 0,519019986 |
| HYG | TP1 (1ng) | 20,59 |  |  |  |
| HYG | AP1 (1ng) | 20,02 |  |  |  |
| HYG | AP1 (1ng) | 20,11 | 20,09 | 56033,07 | 0,720936732 |
| HYG | AP1 (1ng) | 20,12 |  |  |  |
| HYG | TFA7 (1ng) | 20,59 |  |  |  |
| HYG | TFA7 (1ng) | 20,74 | 20,68 | 38765,42 | 0,687386083 |
| HYG | TFA7 (1ng) | 20,72 |  |  |  |
| ZEO | TP1 (1ng) | 16,18 |  |  |  |
| ZEO | TP1 (1ng) | 16,21 | 16,19 | 531283 | 6,635036938 |
| ZEO | TP1 (1ng) | 16,19 |  |  |  |
| ZEO | AP1 (1ng) | 16,33 |  |  |  |
| ZEO | AP1 (1ng) | 16,50 | 16,38 | 469161,3 | 6,036356997 |
| ZEO | AP1 (1ng) | 16,33 |  |  |  |

**Table S5:** The in vitro detoxification of transformants as mg L^-1^ h^-1^ in various enzyme assays.

| Strain | Furfural assay* | Formic acid assay | Cinnamic acid assay |
| --- | --- | --- | --- |
| CelluX^TM^1 | 1.33 | n.d. | n.d. |
| AA6 | 0.67 | - | - |
| TF2 | 2 | n.d. | - |
| PI3 | - | - | 3.93 |
| TFA7 | 1.33 | n.d. | - |
| AP1 | 2.0 | 110 mg L^-1^ | n.d. |
| TP1 | 0.67 | 117 mg L^-1^ | 1.63 |

* No difference relative to control (*p*<0.05)

n.d. – no difference detected
