# Supplementary material for: Rational engineering of Saccharomycescerevisiae towards improved tolerance to multiple inhibitors in lignocellulose fermentations
Source: Biotechnol Biofuels. 2021 Aug 28;14:173. doi: 10.1186/s13068-021-02021-w (PMC8403374; doi:10.1186/s13068-021-02021-w)
Supplement: Supplementary file 2 — Additional file 2: Growth and ethanol yields in round 1 and round 2 transformants. The % increment in growth and ethanol yield relative to parental strain for the 1st round transformants (Figure S1) and 2nd round transformants (Figure S2) in fermentations with 2% SC-X supplemented with 65% v/v sugarcane hydrolysate at 120 h. [file 13068_2021_2021_MOESM2_ESM.docx]

**Rational engineering of *Saccharomyces cerevisiae* towards improved tolerance to multiple inhibitors in lignocellulose fermentations**

Bianca A. Brandt; Maria D.P. García-Aparicio, Johann F. Görgens; Willem H. van Zyl

**Additional file 2: Supplementary figures**


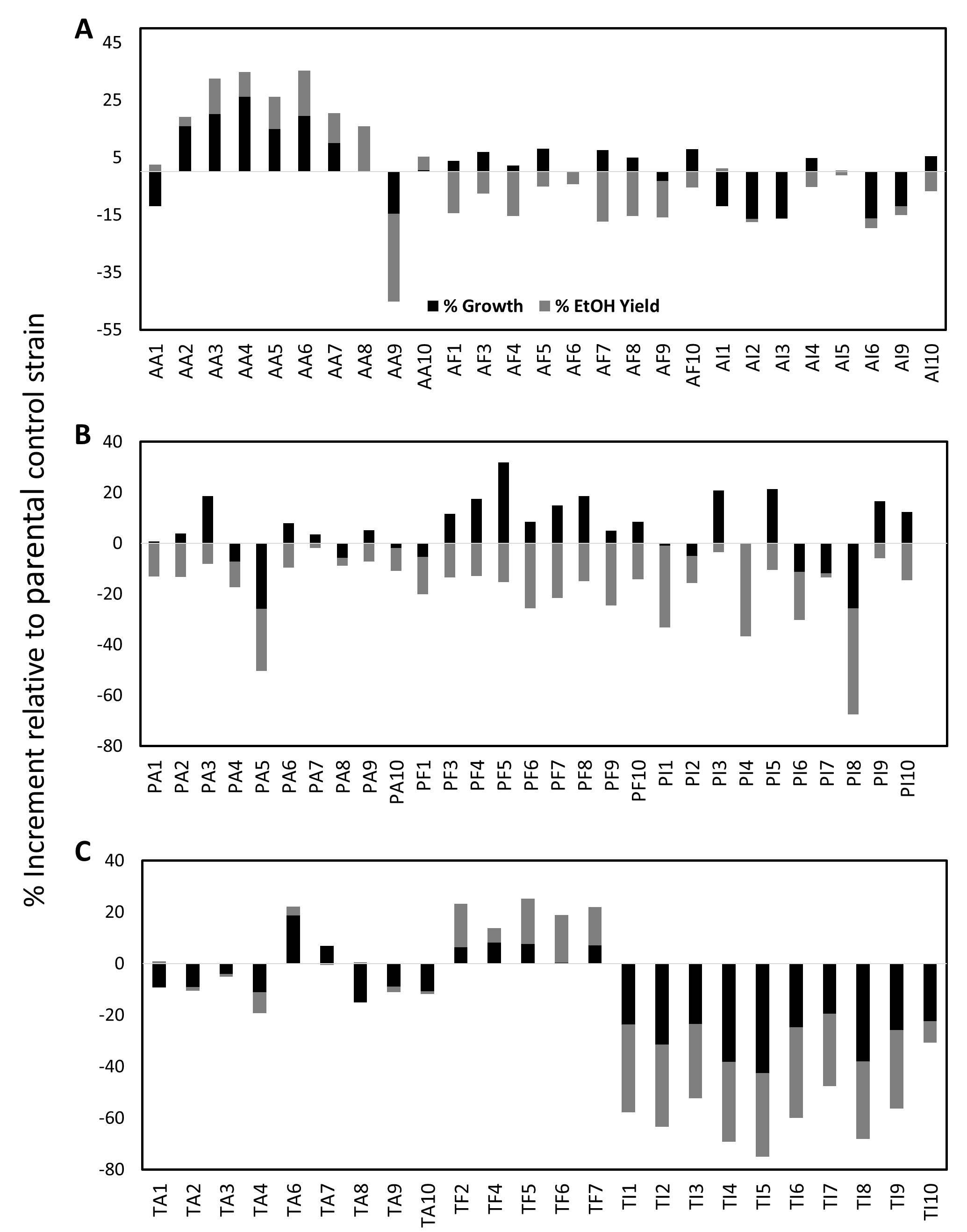


**Figure S1**: The % increment in growth and ethanol yield relative to parental strain for the 1^st^ round transformants for the (A) ARI1-derivatives, (B) PAD1-derivatives and (C) TAL1-derivatives in fermentations in 2% SC-X supplemented with 65% v/v sugarcane hydrolysate at 120 h.


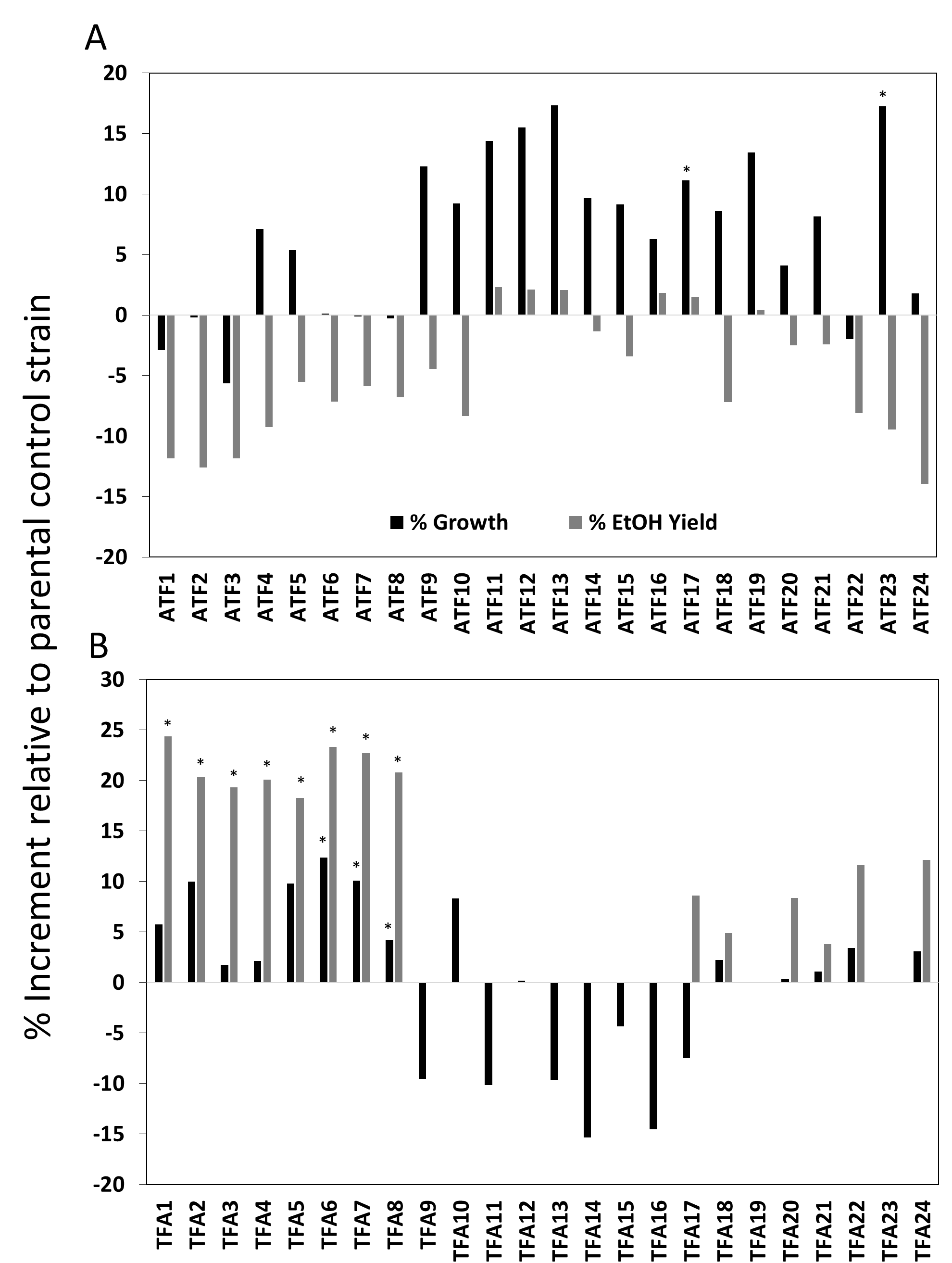


**Figure S2:** The % increment in growth and ethanol yield relative to parental strain for the 2nd round transformants for the (A) AA6 (ARI1+ADH6) + TF (TAL1 + FDH1) transformants, and (B) TF2 (TAL1 + FDH1) + AA transformants in fermentations in 2% SC-X supplemented with 65% v/v sugarcane hydrolysate at 120 h. The asterisks indicates significant differences at p < 0.05.
